# CC16 augmentation reduces exaggerated COPD-like disease in *Cc16*-deficient mice

**DOI:** 10.1172/jci.insight.130771

**Published:** 2023-03-22

**Authors:** Joselyn Rojas-Quintero, Maria Eugenia Laucho-Contreras, Xiaoyun Wang, Quynh-Anh Fucci, Patrick R. Burkett, Se-Jin Kim, Duo Zhang, Yohannes Tesfaigzi, Yuhong Li, Abhiram R. Bhashyam, Zhang Li, Haider Khamas, Bartolome Celli, Aprile L. Pilon, Francesca Polverino, Caroline A. Owen

**Affiliations:** 1 Section of Pulmonary, Critical Care and Sleep Medicine, Department of Medicine, Baylor College of Medicine, Houston, Texas, USA.; 2 Division of Pulmonary and Critical Care Medicine, Brigham and Women’s Hospital and Harvard Medical School, Boston, Massachusetts, USA.; 3 Fundación Neumológica Colombiana, Bogotá, Colombia.; 4 Clinical and Experimental Therapeutics program, College of Pharmacy, University of Georgia and Charlie Norwood VA Medical Center, Augusta, Georgia, USA.; 5 Pulmonary Center, Boston University School of Medicine, Boston, Massachusetts, USA.; 6 Department of Orthopedic Surgery, Massachusetts General Hospital, Boston, Massachusetts, USA.; 7 APCBio Innovations, Inc., Rockville, Maryland, USA.

**Keywords:** Immunology, Inflammation, Innate immunity, NF-kappaB

## Abstract

Low Club Cell 16 kDa protein (CC16) plasma levels are linked to accelerated lung function decline in patients with chronic obstructive pulmonary disease (COPD). Cigarette smoke–exposed (CS-exposed) *Cc16^–/–^* mice have exaggerated COPD-like disease associated with increased NF-κB activation in their lungs. It is unclear whether CC16 augmentation can reverse exaggerated COPD in CS-exposed *Cc16^–/–^* mice and whether increased NF-κB activation contributes to the exaggerated COPD in CS-exposed *Cc16^–/–^* lungs. CS-exposed WT and *Cc16^–/–^* mice were treated with recombinant human CC16 (rhCC16) or an NF-κB inhibitor versus vehicle beginning at the midpoint of the exposures. COPD-like disease and NF-κB activation were measured in the lungs. RhCC16 limited the progression of emphysema, small airway fibrosis, and chronic bronchitis-like disease in WT and *Cc16^–/–^* mice partly by reducing pulmonary inflammation (reducing myeloid leukocytes and/or increasing regulatory T and/or B cells) and alveolar septal cell apoptosis, reducing NF-κB activation in CS-exposed *Cc16^–/–^* lungs, and rescuing the reduced Foxj1 expression in CS-exposed *Cc16^–/–^* lungs. IMD0354 treatment reduced exaggerated lung inflammation and rescued the reduced Foxj1 expression in CS-exposed *Cc16^–/–^* mice. RhCC16 treatment reduced NF-κB activation in luciferase reporter A549 cells. Thus, rhCC16 treatment limits COPD progression in CS-exposed *Cc16^–/–^* mice partly by inhibiting NF-κB activation and represents a potentially novel therapeutic approach for COPD.

## Graphical abstract

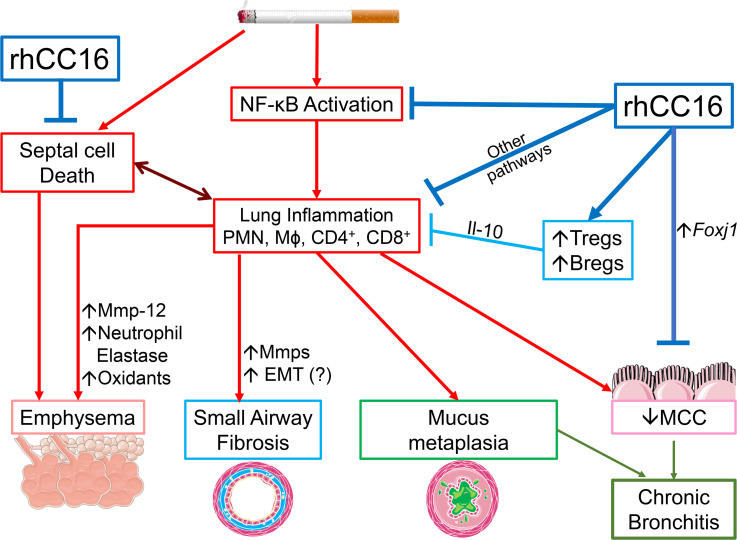

## Introduction

Chronic obstructive pulmonary disease (COPD) is a progressive, debilitating disease characterized by airflow obstruction that is poorly reversible. Currently, there is a lack of medical therapies that significantly alter the course of COPD (1). From 1990 to 2015, the prevalence of COPD increased by 44.2%, and in 2015, it affected approximately 175 million people and was responsible for 3.2 million deaths worldwide (2). Cigarette smoke (CS), the strongest risk factor for COPD in industrialized countries (1–3), induces chronic lung inflammation, oxidative stress, alveolar septal cell apoptosis, and aberrant repair processes (4). COPD patients can develop different pulmonary pathologies including emphysema, small airway fibrosis (SAF), and mucus hypersecretion that vary in severity from patient to patient (3).

Club cell 16 kDa protein (CC16) is a highly conserved, circulating 16 kDa homodimeric protein. The main cellular source of CC16 is airway club cells, which secrete CC16 into the epithelial airway surface liquid from where it diffuses into the circulation (5). *Cc16^–/–^* mice have increased pulmonary inflammation and lung injury in models of inflamm-aging (5), eosinophilic inflammation (6), and viral infection associated with macrophage-driven pulmonary inflammation (7). CC16’s pleiotropic activities are probably due to its binding to various receptors (8).

In large observational cohort studies serum CC16 protein levels are lower in patients with COPD than smokers and nonsmokers (9). Low CC16 plasma levels are linked to COPD progression (10–12) and the chronic bronchitis (CB) phenotype (12). CC16 is one of the most highly hypermethylated genes in small airway epithelial cells in smokers (13), suggesting that reduced airway CC16 expression in smokers and COPD patients is mediated, in part, by epigenetic silencing. Airway CC16 staining is also reduced in CS-exposed mice and nonhuman primates (11, 12). CS-exposed *Cc16^–/–^* mice develop greater lung inflammation, airspace enlargement, mucus metaplasia, and SAF than CS-exposed wild-type (WT) mice (11, 12), and these changes are associated with increased NF-κB activation in the lungs (5, 12). However, whether the increased NF-κB activation detected in the lungs of CS-exposed *Cc16^–/–^* mice directly contributes to their exuberant lung inflammatory response to CS and exaggerated COPD development is unclear.

It has been hypothesized that augmenting CC16 lung levels represents a therapeutic approach for COPD (8). Consistent with this notion, adenovirus-mediated overexpression of Cc16 in the lungs of WT and *Cc16^–/–^* mice protected them from developing CS-induced lung inflammation and airway mucus cell metaplasia (11). Building upon these findings, we tested 2 hypotheses: first, delivering recombinant human CC16 (rhCC16) protein to the lung limits the progression of CS-induced COPD-like parenchymal and airway disease in CS-exposed WT and *Cc16^–/–^* mice, and second, delivering a small molecule NF-κB inhibitor to *Cc16^–/–^* mice reduces their exuberant pulmonary inflammatory response to CS, thereby mechanistically linking the increased NF-κB activation observed in their lungs to their pulmonary phenotype when exposed to CS.

## Results

### COPD-like pulmonary pathologies.

**Figure 1:**
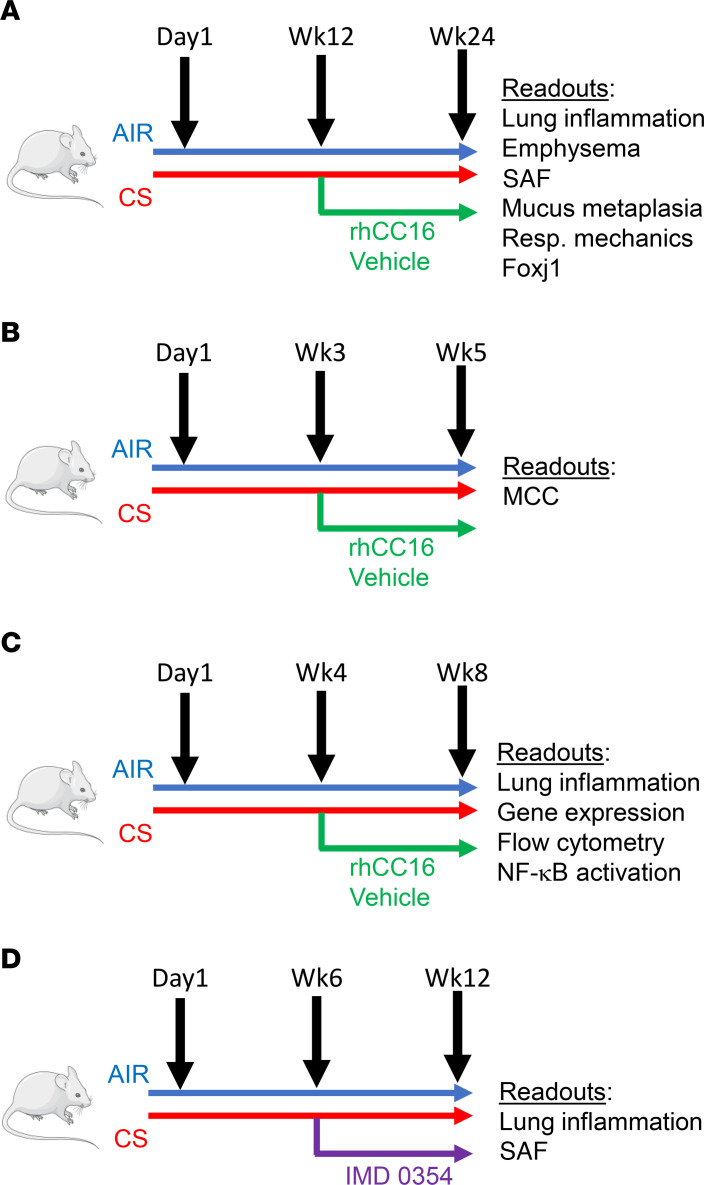
CS exposure and treatment protocols. In **A**, WT and *Cc16^–/–^* mice were exposed to air or CS for a total of 24 weeks, receiving rhCC16 protein weeks 12–24. In **B**, WT and *Cc16^–/–^* mice were exposed to air or CS for a total of 5 weeks, receiving rhCC16 protein from week 3 through week 5. In **C**, WT and *Cc16^–/–^* mice were exposed to air or CS for a total of 8 weeks, receiving rhCC16 protein from week 4 through week 8. In **D**, WT and *Cc16^–/–^* mice were exposed to air or CS for a total of 12 weeks, receiving NF-κB inhibitor from week 6 through week 12. The readouts measured for each exposure are indicated. Foxj1, Forkhead box-J1.

**Figure 2:**
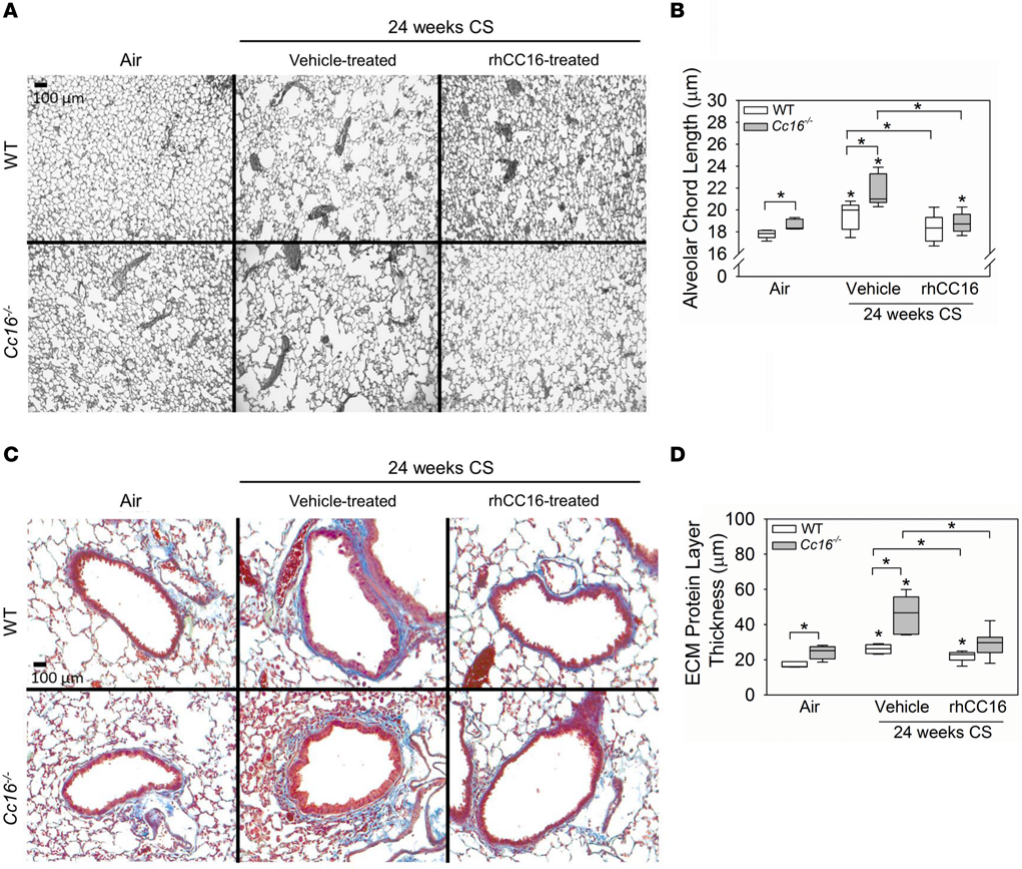
Treating WT and *Cc16^–/–^* mice exposed to CS for 24 weeks with rhCC16 limits the progression of COPD-like disease. WT and *Cc16^–/–^* mice were exposed to air (5–6 mice/group) or CS for 24 weeks (12–13 mice/group), and rhCC16 (75 μg of rhCC16; 6–7 mice/group) or vehicle (6 mice/group) was delivered thrice weekly by the i.n. route to CS-exposed mice for the last 12 weeks of the CS exposures. Sections of inflated and fixed lungs were stained with either Gill’s stain (**A**) and alveolar chord lengths as a measure of airspace size were quantified (**B**) or with Masson’s trichrome stain (**C**, which stains extracellular matrix [ECM] proteins blue) and the thickness of the layer of ECM deposited around the small airways was quantified (**D**). In **B** and **D**, boxes in the box plots show the medians and 25th and 75th percentiles, and whiskers show the 10th and 90th percentiles. Data were analyzed using 1-way ANOVAs followed by pairwise testing with Mann-Whitney *U* tests. Asterisks indicate *P* < 0.05 vs. air-exposed mice belonging to the same genotype or the group indicated.

To evaluate the effects of rhCC16 on COPD-like lung disease in mice, WT and *Cc16^–/–^* mice were exposed to CS or room air for 24 weeks to induce emphysema and SAF (14) (Figure 1A). CS-exposed mice were treated thrice weekly with rhCC16 or vehicle delivered by the intranasal (i.n.) route beginning at the midpoint of the CS exposures. CS-exposed and vehicle-treated *Cc16^–/–^* mice had greater emphysema (Figure 2, A and B) and SAF (Figure 2, C and D) than CS-exposed and vehicle-treated WT mice, as expected (10). Treating CS-exposed WT and *Cc16^–/–^* mice with rhCC16 abrogated both emphysema and SAF in both genotypes. Healthy male mice have lower circulating Cc16 levels due to higher glomerular filtration rates (15). There were no significant differences in emphysema or SAF according to sex (Supplemental Table 1; supplemental material available online with this article; https://doi.org/10.1172/jci.insight.130771DS1) or in respiratory mechanics between CS-exposed and rhCC16-treated and CS-exposed and vehicle-treated WT or *Cc16^–/–^* mice (Supplemental Table 2).

**Figure 3:**
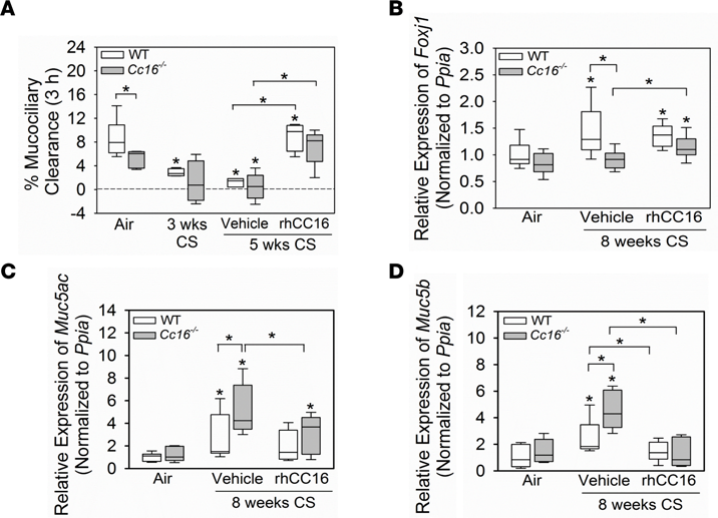
Treatment with rhCC16 limits the progression of CB-like disease in WT and *Cc16^–/–^* mice exposed for CS for up to 8 weeks. In **A**, WT and *Cc16^–/–^* mice were exposed to air (5–8 mice/group) or CS (4 mice/group) for 3 weeks, which is sufficient to impair mucociliary clearance (MCC) in WT mice (16). Other cohorts of mice were exposed to CS for 3 weeks, and then thrice weekly treatment with rhCC16 (75 μg of rhCC16; 4–5 mice/group) or vehicle (3–8 mice/group) delivered by the i.n. route was initiated and continued during an additional 2 weeks of CS exposure. ^99m^Tc-sc was instilled into the lungs of anesthetized mice, and the efficiency of ^99m^Tc-sc clearance from lungs was measured using noninvasive, 3-dimensional μ-SPECT imaging. In **B**–**D**, WT and *Cc16^–/–^* mice were exposed to air (7 mice/group) or CS for 8 weeks (15–17 mice/group). CS-exposed mice were treated thrice weekly with rhCC16 (8 mice/group) or vehicle (7–9 mice/group) for the last 4 weeks of the 8-week CS exposures. The expression of *Foxj1* (**B**), *mucin 5ac* (*Muc5ac*; **C**), and *Muc5b* (**D**) was measured in whole lung samples using reverse transcription quantitative real-time PCR (RT-qPCR). In **A**–**D**, boxes in the box plots show the medians and 25th and 75th percentiles, and the whiskers show the 10th and 90th percentiles. Data were analyzed using 1-way ANOVAs followed by pairwise testing with Mann-Whitney *U* tests. Asterisks indicate *P* < 0.05 vs. air-exposed mice belonging to the same genotype or the group indicated.

Impaired mucociliary clearance (MCC) and mucus hypersecretion contribute to the CB COPD phenotype. Exposing WT mice to CS for 3 weeks is sufficient to almost completely inhibit MCC (16). However, to our knowledge, MCC has not been evaluated previously in air-exposed *Cc16^–/–^* mice or CS-exposed WT versus *Cc16^–/–^* mice. WT and *Cc16^–/–^* mice were exposed to air or CS for 3 weeks, and MCC was measured by delivering ^99m^technetium-sulfur colloid (^99m^Tc-sc) to the lungs and measuring its clearance using 3D μ-SPECT imaging (Figure 1B). Exposing WT mice to CS for 3 weeks severely impaired MCC (Figure 3A) (16). *Cc16^–/–^* mice had impaired MCC even at baseline, and exposing them to CS for 3 weeks further impaired MCC (Figure 3A). WT and *Cc16^–/–^* mice were exposed to CS for 3 weeks and treated with rhCC16 or vehicle for an additional 2 weeks of CS exposure (Figure 1B). Treating CS-exposed WT and *Cc16^–/–^* mice with rhCC16 restored MCC in both genotypes (Figure 3A).

Effective MCC requires motile cilia on airway epithelial cells, and Foxj1 is a transcription factor required for the expression of genes involved in ciliary motility (17, 18). To determine the effect of CS exposure and rhCC16 treatment on *Foxj1* expression, mice were exposed to CS for 8 weeks, and thrice weekly rhCC16 or vehicle treatment was initiated at the midpoint of these exposures and continued for the second 4 weeks (Figure 1C). CS-exposed and vehicle-treated WT (but not *Cc16^–/–^*) mice had increased *Foxj1* expression in their lungs compared with the air-exposed corresponding controls (Figure 3B). RhCC16 treatment increased *Foxj1* expression in CS-exposed *Cc16^–/–^* mice versus vehicle-treated *Cc16^–/–^* mice but did not increase *Foxj1* expression further in CS-exposed WT mice.

**Figure 4:**
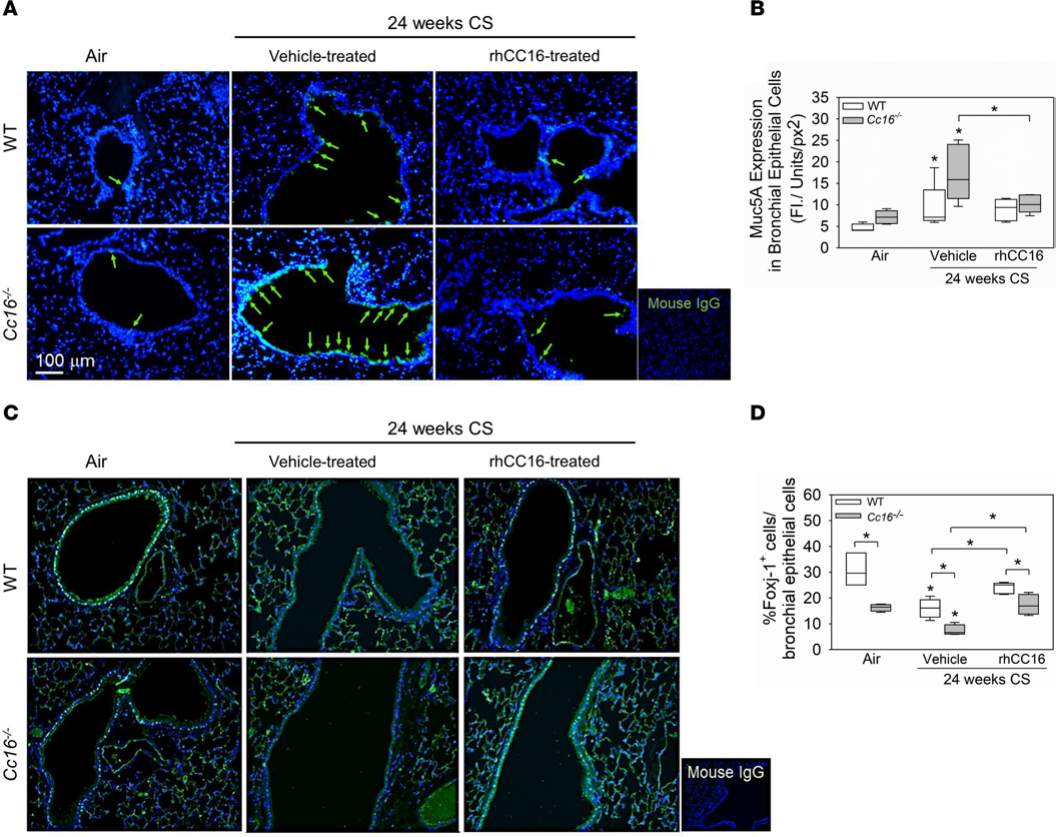
Treatment of mice with rhCC16 limits the development of mucus cell metaplasia and rescues Foxj1 expression in mice exposed chronically to CS. WT and *Cc16^–/–^* mice were exposed to air (3–5 mice/group) or CS for 24 weeks (10–13 mice/group), and rhCC16 (75 μg of rhCC16; 5–7 mice/group) or vehicle (5–6 mice/group) was delivered thrice weekly by the i.n. route to CS-exposed mice for the last 12 weeks of the CS exposures. Lung sections were immunostained for Muc5ac (**A** and **B**) and Foxj1 (**C** and **D**), and the percentage of positively stained airway epithelial cells was determined. Arrows point to Muc5ac-positive cells. The box plots show the medians and 25th and 75th percentiles, and whiskers show the 10th and 90th percentiles. Data were analyzed using 1-way ANOVAs followed by pairwise testing with Mann-Whitney *U* tests. Asterisks indicate *P* < 0.05 vs. air-exposed mice belonging to the same genotype or the group indicated. FI, fluorescence Intensity; px, pixel.

To evaluate the effects of rhCC16 treatment on airway mucus metaplasia, WT and *Cc16^–/–^* mice were exposed to CS for 4 weeks to induce airway mucus metaplasia (19), followed by thrice weekly treatment with rhCC16 or vehicle for an additional 4 weeks of CS exposure (Figure 1C). CS-exposed and vehicle-treated *Cc16^–/–^* mice had greater increases in *mucin 5ac* (*Muc5ac*) and *Muc5b* gene expression levels in their lungs than CS-exposed and vehicle-treated WT mice (Figure 3, C and D). RhCC16 treatment abrogated CS-induced increases in *Muc5ac* and *Muc5b* gene expression levels in CS-exposed WT and *Cc16^–/–^* mice. *Muc1* expression levels were similar across experimental groups (Supplemental Figure 1). WT and *Cc16^–/–^* mice were then exposed chronically to CS for 24 weeks and treated with rhCC16 solution or vehicle for the second 12 weeks of the 24-week CS exposure (Figure 1A). Muc5ac staining was robustly increased in CS-exposed and vehicle-treated *Cc16^–/–^* and *WT* mice versus air-exposed mice belonging to the same genotype. Muc5ac staining was significantly lower in rhCC16-treated versus vehicle-treated CS-exposed *Cc16^–/–^* mice (Figure 4, A and B).

As rhCC16 treatment rescued the reduced *Foxj1* expression in acute CS exposures, we quantified Foxj1 positively stained cells in the 24-week CS exposure model (Figure 1A). Air-exposed *Cc16^–/–^* mice had lower numbers of Foxj1 positively stained airway epithelial cells than air-exposed WT mice. CS-exposed and vehicle-treated WT and *Cc16^–/–^* mice had a lower number of Foxj1 positively stained cells than air-exposed mice belonging to the same genotype. CS-exposed and vehicle-treated *Cc16^–/–^* mice had lower numbers of Foxj1 positively stained cells than CS-exposed and vehicle-treated WT mice. RhCC16 treatment increased the number of Foxj1 positively stained airway epithelial cells in both WT and *Cc16^–/–^* mice (Figure 4, C and D).

CS exposure impairs weight gain in WT mice (14). Air-exposed WT and *Cc16^–/–^* mice showed progressive increases in body weight over 24 weeks (Supplemental Figure 2A). CS-exposed and vehicle-treated WT mice (Figure 1A) had reduced weight gain during weeks 12–24 versus air-exposed WT mice; CS-exposed and rhCC16-treated WT mice had similar weight gain as the air-exposed controls (Supplemental Figure 2A). CS-exposed and vehicle-treated *Cc16^–/–^* mice had similar weight gain as air-exposed controls (Supplemental Figure 2B). CS-exposed and rhCC16-treated *Cc16^–/–^* mice had greater increases in body weight from the 18th week of CS exposure onward compared with air-exposed and also CS-exposed and vehicle-treated *Cc16^–/–^* mice (Supplemental Figure 2B).

### Mechanistic studies.

CC16 has potent antiinflammatory activities in the CS-exposed lung (5, 8, 10). Pulmonary inflammation contributes to emphysema development, SAF, and airway mucus hypersecretion (4). We investigated the effects of rhCC16 on CS-induced acute and chronic pulmonary inflammation, alveolar septal cell apoptosis, and impaired septal cell proliferation, which also contribute to emphysema development (10, 19).

### Lung inflammation.

**Figure 5:**
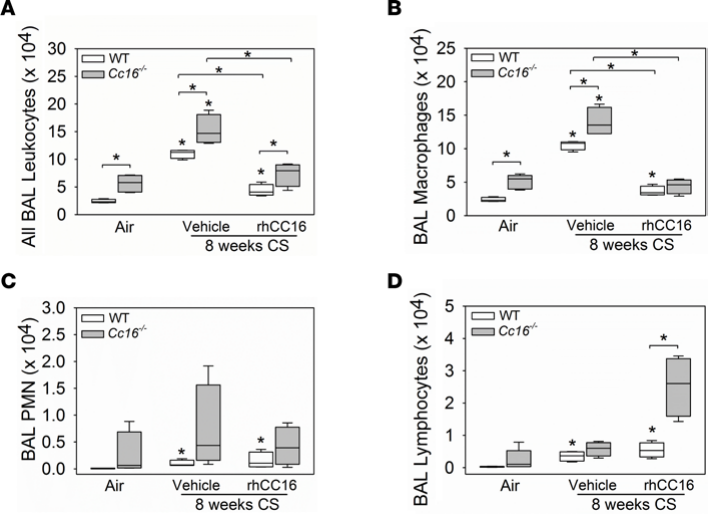
Treating CS-exposed WT and *Cc16^–/–^* mice with rhCC16 abrogates acute pulmonary inflammation. WT and *Cc16^–/–^* mice were exposed to air (4–5 mice/group) or CS for 8 weeks (8 mice/group). CS-exposed mice were treated thrice weekly with rhCC16 (75 μg of rhCC16; 4 mice/group) or vehicle (4 mice/group) for the last 4 weeks of the exposures, and bronchoalveolar lavage (BAL) was performed. BAL total leukocytes (**A**), macrophages (**B**), PMNs (**C**), and lymphocytes (**D**) were counted. Boxes in the box plots show the median values and 25th and 75th percentiles, and whiskers show the 10th and 90th percentiles. Data were analyzed using 1-way ANOVAs followed by pairwise testing with Mann-Whitney *U* tests. Asterisks indicate *P* < 0.05 vs. air-exposed mice belonging to the same genotype or the group indicated.

WT and *Cc16^–/–^* mice were exposed to CS for 4 weeks to induce acute pulmonary inflammation (10). Treatment with rhCC16 versus vehicle was then initiated in CS-exposed mice via the i.n. route and continued thrice weekly for an additional 4 weeks of CS exposure, and then pulmonary inflammation was quantified (Figure 1C). CS induced greater increases in bronchoalveolar lavage (BAL) total leukocyte and macrophage counts in *Cc16^–/–^* versus WT mice (Figure 5, A and B). RhCC16 treatment abrogated CS-induced increases in BAL total leukocyte and macrophage counts in both genotypes (Figure 5, A and B). CS exposure modestly increased BAL polymorphonuclear neutrophil (PMN) counts in WT (but not *Cc16^–/–^* mice); rhCC16 treatment had no effect on BAL PMN counts in CS-exposed WT mice (Figure 5C). CS-exposed WT mice had higher BAL lymphocyte counts than air-exposed WT mice. RhCC16-treated and CS-exposed *Cc16^–/–^* mice (but not WT mice) had a striking increase in BAL lymphocyte counts versus CS-exposed and vehicle-treated control mice (Figure 5D).

**Figure 6:**
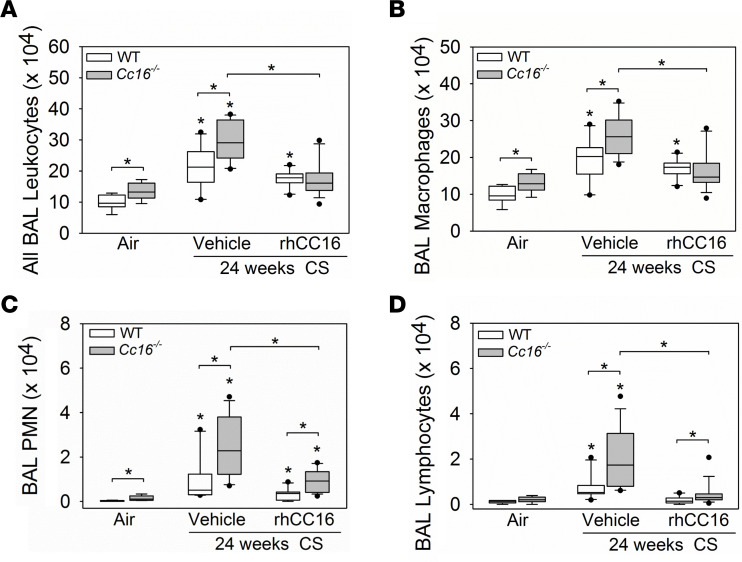
Treating CS-exposed *Cc16^–/–^* mice with rhCC16 reduces chronic pulmonary inflammation. WT and *Cc16^–/–^* mice were exposed to air (7–8 mice/group) or CS for 24 weeks (20–25 mice/group). CS-exposed mice were treated with rhCC16 (75 μg of rhCC16; 10–15 mice/group) or vehicle (10–13 mice/group) thrice weekly for the last 12 weeks of the exposures and BAL was performed. BAL total leukocytes (**A**), macrophages (**B**), PMNs (**C**), and lymphocytes (**D**) were counted. Boxes in the box plots show the median values and 25th and 75th percentiles, and whiskers show the 10th and 90th percentiles. Data were analyzed using 1-way ANOVAs followed by pairwise testing with Mann-Whitney *U* tests. Asterisks indicate *P* < 0.05 vs. air-exposed mice belonging to the same genotype or the group indicated.

WT and *Cc16^–/–^* mice were exposed to CS for 12 weeks to induce chronic pulmonary inflammation (10). Treatment with rhCC16 or vehicle was then initiated in the CS-exposed mice via the i.n. route and continued thrice weekly during an additional 12 weeks of CS exposure, and pulmonary inflammation was quantified (Figure 1A). CS-exposed and vehicle-treated *Cc16^–/–^* mice had greater increases in BAL total leukocyte and leukocyte subset counts than CS-exposed and vehicle-treated WT mice compared with the air-exposed controls (Figure 6, A–D). Treating *Cc16^–/–^* mice (but not WT mice) with rhCC16 substantially abrogated the CS-induced increases in BAL total leukocyte, macrophage, PMN, and lymphocyte counts versus vehicle-treated control mice (Figure 6, A–D). CS-exposed and vehicle-treated WT and *CC16^–/–^* mice had increased BAL total leukocyte and leukocyte subset counts in both sexes in both genotypes, and rhCC16 treatment reduced CS-induced increases in BAL total leukocyte and leukocyte subset counts in both sexes in both genotypes (Supplemental Table 3).

**Figure 7:**
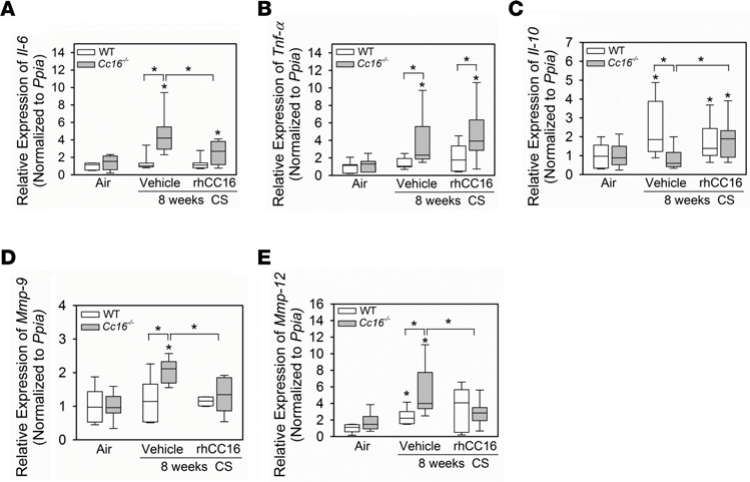
Treating CS-exposed *Cc16^–/–^* mice with rhCC16 reduces the expression of some mediators of inflammation and injury in their lungs. WT and *Cc16^–/–^* mice were exposed to air (7 mice/group) or CS for 8 weeks (15–17 mice/group). CS-exposed mice were treated thrice weekly with rhCC16 (8 mice/group) or vehicle (7–9 mice/group) for the last 4 weeks of the 8-week CS exposures. The expression of *Il-6* (**A**), *Tnf-α* (**B**), *Il-10* (**C**), *Mmp-9* (**D**), and *Mmp-12* (**E**) was measured in whole lung samples using RT-qPCR. Boxes in the box plots show the medians and 25th and 75th percentiles, and whiskers show the 10th and 90th percentiles. Data were analyzed using 1-way ANOVAs followed by pairwise testing with Mann-Whitney *U* tests. Asterisks indicate *P* < 0.05 vs. air-exposed mice belonging to the same genotype or the group indicated.

CS-exposed and vehicle-treated *Cc16^–/–^* (but not WT) mice had increased expression of *Il-6* and *Tnf-α* in their lungs (Figure 7, A and B). RhCC16 treatment reduced the expression of *Il-6* (but not *Tnf-α*) in the lungs of CS-exposed *Cc16^–/–^* mice. CS-exposed and vehicle-treated WT (but not *Cc16^–/–^*) mice had increased expression of antiinflammatory *Il-10* in their lungs. RhCC16 treatment increased *Il-10* expression in the lungs of CS-exposed *Cc16^–/–^* (but not WT) mice versus that in CS-exposed and vehicle-treated control mice (Figure 7C). CS-exposed and vehicle-treated WT (but not *Cc16^–/–^*) mice had reduced *Tgf-β* expression in their lungs, but rhCC16 treatment had no effect on *Tgf-β* expression in the lungs of CS-exposed mice (Supplemental Figure 3A). CS-exposed and vehicle-treated *Cc16^–/–^* (but not WT) mice had increased expression of matrix metalloproteinase-9 (*Mmp-9*), and CS-exposed and vehicle-treated WT and *Cc16^–/–^* mice had increased expression of *Mmp-12* in their lungs versus air-exposed controls. RhCC16 treatment to CS-exposed *Cc16^–/–^* (but not WT) mice reduced *Mmp-9* and *Mmp-12* gene expression in their lungs versus CS-exposed and vehicle-treated mice (Figure 7, D and E). CS-exposed and vehicle-treated WT mice had increased expression of *Ccl-2* in their lungs, and CS-exposed and vehicle-treated WT and *Cc16^–/–^* had increased expression of *Ccl-3* and *Ccl-5* in their lungs versus air-exposed control mice. However, rhCC16 treatment did not alter the expression of any of these chemokines in the lungs of CS-exposed mice (Supplemental Figure 3, B–D).

### Lymphocyte subsets.

As rhCC16 treatment increased lymphocyte counts and *Il-10* expression in the lungs of CS-exposed *Cc16^–/–^* mice (Figure 5D and Figure 7C), the relative frequencies of lung lymphocyte subsets were quantified including those that produce IL-10. WT and *Cc16^–/–^* mice were exposed to air or CS for 8 weeks, and CS-exposed mice were treated with rhCC16 or vehicle for the last 4 weeks of the exposures. Enzymatic lung digests were immunostained for markers of lymphocyte subsets (20) and markers of activation, including programmed cell death 1 (PD-1) (21), T cell Ig domain and mucin domain protein-3 (Tim-3) (22), T cell immunoreceptor with Ig and ITIM domains (TIGIT) (23), and suppression of tumorigenicity-2 (ST2) (24).

The frequencies of CD4^+^ and CD8^+^ T cells were increased in the lungs of air-exposed *Cc16^–/–^* versus WT mice (Table 1). CS-exposed and vehicle-treated WT (but not *Cc16^–/–^*) mice had an increased frequency of lung-infiltrating CD4^+^ and CD8^+^ T cells. RhCC16 treatment did not alter CD4^+^ or CD8^+^ T cell frequencies in either CS-exposed WT or *Cc16^–/–^* mice versus CS-exposed and vehicle-treated controls. CS-exposed and vehicle-treated WT and *Cc16^–/–^* mice did not differ in their frequencies of lung-resident type 2 ILCs (ST2^+^Thy1^+^CD4^–^ cells), NK cells, or CD19^+^ B cells, and rhCC16 treatment did not alter the frequency of these cells in CS-exposed lungs of either genotype (Table 1).

CS-exposed and vehicle-treated *Cc16^–/–^* (but not WT) mice had an increased frequency of PD-1^+^CD4^+^ T cells versus air-exposed controls, but there were no differences in PD-1 expression on CD8^+^ T cells or B cells between genotypes (Supplemental Figure 4, A–C). CS-exposed and vehicle-treated *Cc16^–/–^* (but not WT) mice had increased lung Tim-3^+^CD4^+^ and Tim-3^+^CD8^+^ T lymphocyte counts versus air-exposed control mice (Supplemental Figure 4, D and E). *Cc16^–/–^* mice had greater lung Tim-3^+^ NK cell counts than WT mice when exposed to air or CS (Supplemental Figure 4F). RhCC16 treatment increased lung PD-1^+^CD8^+^ T cell counts only in CS-exposed *Cc16^–/–^* mice versus air-exposed controls but had no effect on lung PD-1^+^CD4^+^ T cell, Tim-3^+^CD4^+^ T cell, Tim-3^+^CD8^+^ T cell, or Tim-3^+^ NK cell counts. Overall, rhCC16 treatment does not exert its protective effects on the CS-exposed lung by promoting the expression of inhibitory cell surface receptors on T or B lymphocytes.

**Figure 8:**
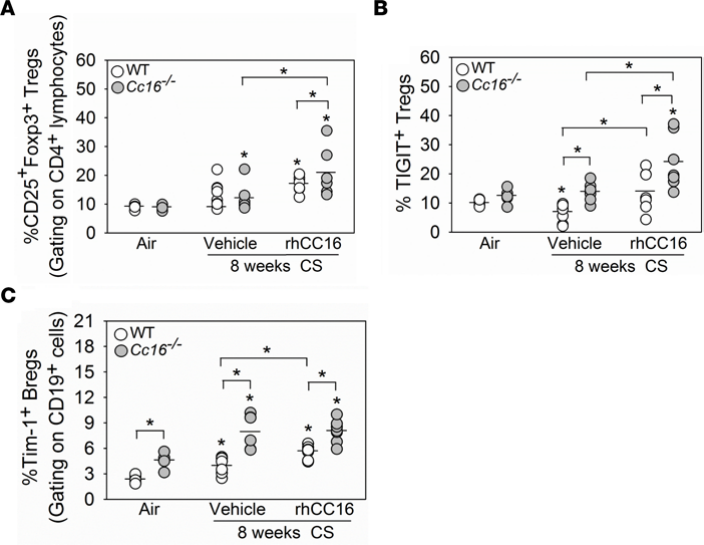
Treating CS-exposed WT and/or *Cc16^–/–^* mice with rhCC16 increases T cell regulatory and/or B regulatory subset counts in their lungs. WT and *Cc16^–/–^* mice were exposed to air (5 mice/group) or CS for 8 weeks (14–15 mice/group), and CS-exposed mice were treated with rhCC16 (75 μg of rhCC16; 7 mice/group) or vehicle (7–8 mice-group) thrice weekly for the last 4 weeks of the exposures. Lymphocyte subsets were quantified in enzymatic lung digests using flow cytometry after immunostaining the cells for: CD25^+^Foxp3^+^ Tregs (gating on CD4^+^ lymphocytes) in **A**, TIGIT^+^ Tregs (gating on CD4^+^CD25^+^Foxp3^+^ lymphocytes) in **B**, and Tim-1^+^ Bregs (gating on CD19^+^ cells) in **C**. The horizontal bars in the dot plots represent the mean values. Data were analyzed using 1-way ANOVAs followed by pairwise testing with Mann-Whitney *U* tests. Asterisks indicate *P* < 0.05 vs. air-exposed mice belonging to the same genotype or the group indicated.

CD25^+^Foxp3^+^ regulatory T cells (Tregs) release IL-10 and negatively regulate effector T cell activity. CS-exposed and vehicle-treated *Cc16^–/–^* (but not WT) mice had an increased frequency of lung-resident Tregs versus air-exposed controls (Figure 8A). RhCC16 treatment increased Treg frequency to a greater extent in CS-exposed *Cc16^–/–^* versus WT lungs. IL-33–expanded Tregs express ST2 (IL-33 receptor) and ST2^+^ Tregs are key suppressors of detrimental Th1 and Th2 immune responses (25). CS-exposed and vehicle-treated *Cc16^–/–^* (but not WT) mice had an increased ST2^+^ Treg frequency in their lungs versus air-exposed control mice. RhCC16 treatment did not alter lung ST2^+^ Treg frequencies in CS-exposed WT or *Cc16^–/–^* mice versus CS-exposed and vehicle-treated control mice belonging to the same genotype (Supplemental Figure 4G). TIGIT^+^ Tregs suppress Th1 and Th17 lymphocyte responses (24, 25). CS-exposed and vehicle-treated WT (but not *Cc16^–/–^*) mice had a reduced frequency of lung TIGIT^+^ Tregs versus air-exposed mice. CS-exposed and vehicle-treated *Cc16^–/–^* mice had a higher frequency of lung TIGIT^+^ Tregs than CS-exposed and vehicle-treated WT mice (Figure 8B). RhCC16 treatment increased the frequency of lung TIGIT^+^ Tregs to a greater extent in CS-exposed *Cc16^–/–^* versus WT mice.

Regulatory B cells (Bregs) (Tim-1^+^ B cells; ref. 26) also secrete IL-10 and negatively regulate T effector cells. Breg frequencies were higher in air-exposed *Cc16^–/–^* versus WT lungs (Figure 8C). CS-exposed and vehicle-treated WT and *Cc16^–/–^* mice had increases in lung Breg frequencies compared with those in air-exposed mice belonging to the same genotype. CS-exposed and vehicle-treated *Cc16^–/–^* mice had increased lung Breg frequencies versus CS-exposed and vehicle-treated WT mice. RhCC16 treatment increased lung Breg frequency only in CS-exposed WT mice versus CS-exposed and vehicle-treated controls (Figure 8C). Thus, rhCC16 may protect the lung from CS-induced injury, in part, by increasing Treg and Breg accumulation in the lungs.

### Alveolar septal cell phenotypes.

**Figure 9:**
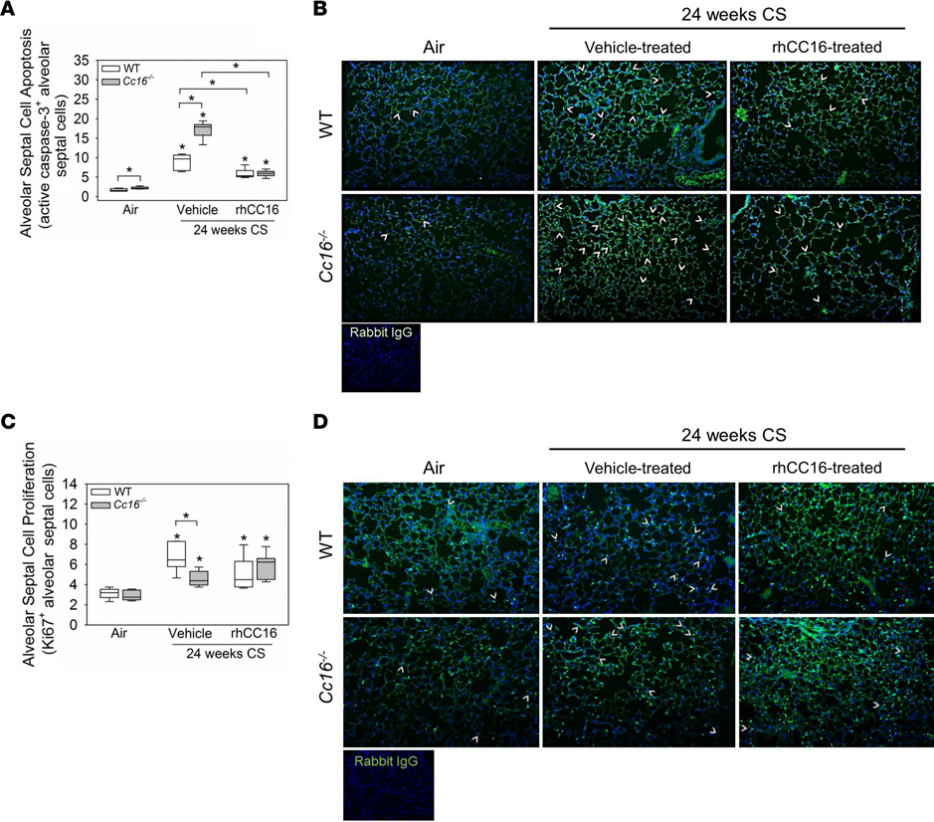
Treating CS-exposed WT and *Cc16^–/–^* mice with rhCC16 reduces alveolar septal cell apoptosis but not proliferation in their lungs. WT and *Cc16^–/–^* mice were exposed to air (6–7 mice/group) or CS for 24 weeks (12–13 mice/group), and CS-exposed mice were treated with rhCC16 (75 μg of rhCC16; 6–7 mice/group) or vehicle (6 mice/group) for thrice weekly the last 12 weeks of the exposures. Inflated lung sections were immunostained for markers of alveolar septal cell apoptosis (active caspase-3; **A** and **B**) or proliferation (Ki67; **C** and **D**). Arrows point to active caspase-3–positive cells (**B**) or Ki67-positive cells (**D**). Boxes in the box plots show the medians and 25th and 75th percentiles, and whiskers show the 10th and 90th percentiles. Data were analyzed using 1-way ANOVAs followed by pairwise testing with Mann-Whitney *U* tests. Asterisks indicate *P* < 0.05 vs. air-exposed mice belonging to the same genotype or the group indicated.

Air-exposed *Cc16^–/–^* mice had modestly increased alveolar septal cell apoptosis (active caspase-3–positive cells) versus air-exposed WT mice (Figure 9A). CS-exposed and vehicle-treated *Cc16^–/–^* mice had greater increases in alveolar septal cell apoptosis than CS-exposed and vehicle-treated WT mice versus air-exposed control mice. RhCC16 treatment abrogated the CS-induced increases in alveolar septal cell apoptosis in both genotypes. CS-exposed and vehicle-treated *Cc16^–/–^* mice had lower increases in alveolar septal cell proliferation (Ki67-positive cells) than CS-exposed and vehicle-treated WT mice (Figure 9, C and D). RhCC16 treatment did not increase alveolar septal cell proliferation in either genotype versus CS-exposed and vehicle-treated controls (Figure 9, A and B). The expression of markers of senescence (*p21*, *p16*, and *Sirtuin*) and activation of the β-catenin/Wnt repair pathway (assessed as expression of *Wisp-2* or *Tcf-7*) was similar in all groups (Supplemental Figure 5, A–E).

**Figure 10:**
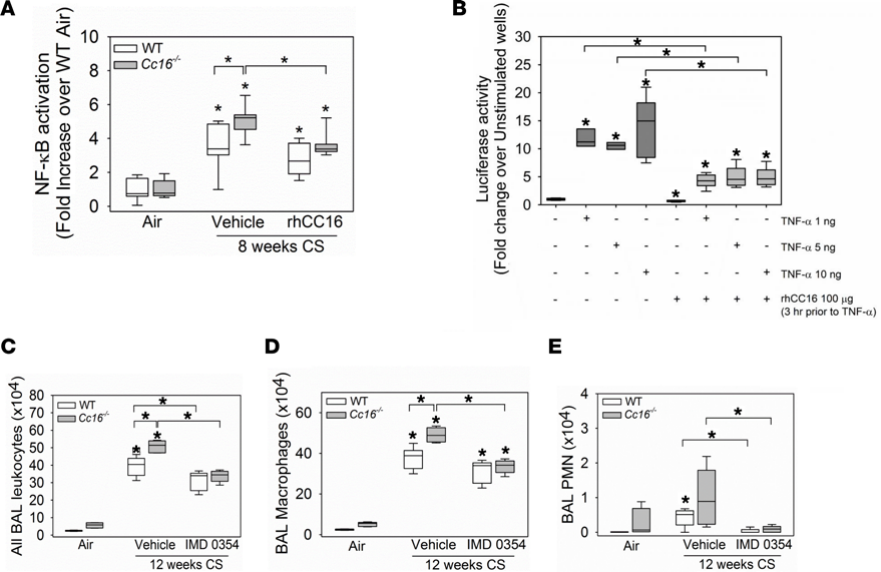
Treating CS-exposed *Cc16^–/–^* mice with rhCC16 reduces NF-κB activation in their lungs, and increased NF-κB activation in the lungs of CS-exposed *Cc16^–/–^* mice contributes to their exaggerated pulmonary inflammatory response to CS. In **A**, WT and *Cc16^–/–^* mice were exposed to air (6 mice/group) or CS for 8 weeks (6–7 mice/group), and CS-exposed mice were treated thrice weekly with rhCC16 (75 μg of rhCC16) or vehicle. NF-κB that translocated to the nucleus was quantified in nuclear extracts of whole lung samples using a TransAM NF-κB kit. In **B**, NF-κB luciferase reporter A549 cells were grown to at least 80% confluence and preincubated for 3 hours with 100 μg/mL of rhCC16. Cells were then activated with rhTNF-α (1–10 ng/mL), and 8 hours later, the cells were lysed and luciferase activity was measured. In **C**, WT and *Cc16^–/–^* mice were exposed to air (3–5 mice/group) or CS for 12 weeks (10–12 mice/group), and CS-exposed mice were treated once daily with a solution of IMD0354 (6 mg/kg body weight) or vehicle via the intraperitoneal route during the last 6 weeks of the CS exposures (5–6 mice/group). BAL was performed, and BAL total leukocytes (**C**), macrophages (**D**), and PMNs (**E**) were counted. Boxes in the box plots show the medians and 25th and 75th percentiles, and whiskers show the 10th and 90th percentiles. Data were analyzed using 1-way ANOVAs followed by pairwise testing with Mann-Whitney *U* tests. In **A** and **C**–**E**, asterisks indicate *P* < 0.05 vs. air-exposed mice belonging to the same genotype or the group indicated; in **B**, asterisks indicate *P* < 0.05 vs. negative control in lane 1 or the group indicated.

The increased pulmonary inflammatory response to CS in *Cc16^–/–^* mice is associated with increased activation of NF-κB in their lungs (10). Thus, we determined whether rhCC16 reduces NF-κB activation in CS-exposed WT and/or *Cc16^–/–^* lungs. Mice were exposed to CS for 4 weeks to induce an acute pulmonary inflammatory response, and treatment with rhCC16 or vehicle was then initiated via the i.n. route and continued during an additional 4 weeks of CS exposure (Figure 1D). NF-κB translocation to the nucleus was quantified in nuclear extracts of whole lung samples. CS-exposed and vehicle-treated *Cc16^–/–^* mice had greater increases in NF-κB activation in their lungs than CS-exposed and vehicle-treated WT mice versus NF-κB activation levels in the lungs of air-exposed control mice belonging to the same genotype (Figure 10A). RhCC16 treatment decreased NF-κB activation only in CS-exposed *Cc16^–/–^* lungs. To provide additional assurance that rhCC16 reduces activation of NF-κB, we conducted an in vitro experiment with NF-κB luciferase reporter A549 cells activated with CS extract (CSE) or TNF-α. TNF-α triggered activation of NF-κB in the reporter assay (Figure 10B), but CSE tested at 1%–10% concentrations did not (Supplemental Figure 6). When rhCC16 was added to cells 3 hours before the TNF-α, rhCC16 reduced TNF-α–induced activation of NF-κB (Figure 10B).

### NF-κB inhibition with a small molecule inhibitor.

**Figure 11:**
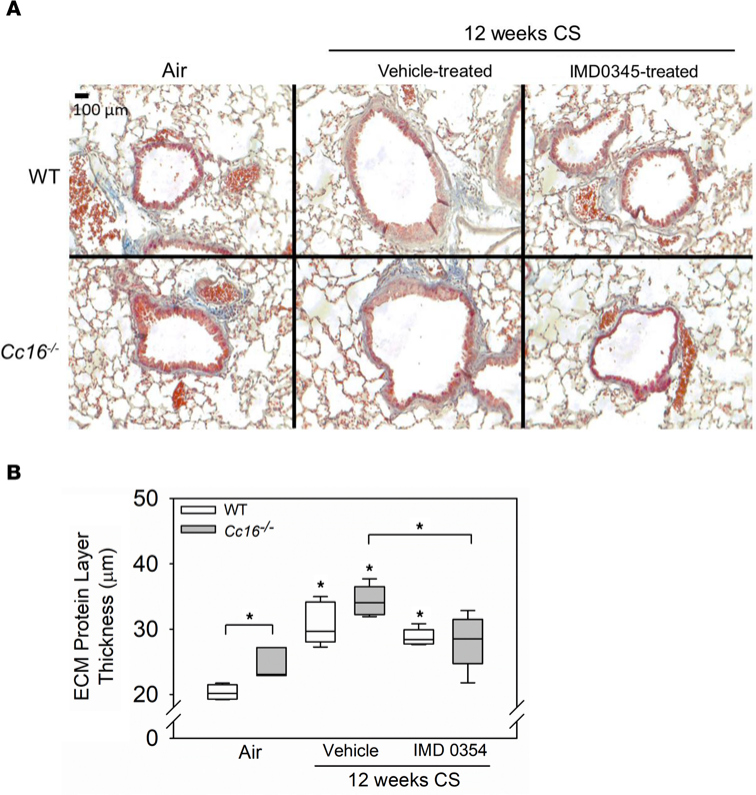
Treating CS-exposed WT and *Cc16^–/–^* mice with IMD0354 reduces mucus metaplasia. In **A** and **B**, WT and *Cc16^–/–^* mice were exposed to air (3–5 mice/group) or CS for 12 weeks (10–12 mice/group), and CS-exposed mice were treated once daily with a solution of IMD0354 (6 mg/kg body weight; 5–6 mice/group) or vehicle via the intraperitoneal route during the last 6 weeks of the CS exposures (5–6 mice/group). Inflated lung sections with Masson’s trichrome stain (**A**), which stains ECM proteins blue. The thickness of the layer of ECM deposited around the small airways was quantified (**B**). The IMD0354-treated *Cc16^–/–^* mice had significantly lower ECM deposit compared with vehicle-treated *Cc16^–/–^* mice (*P* = 0.0124). Boxes in the box plots show the medians and 25th and 75th percentiles, and whiskers show the 10th and 90th percentiles. Data were analyzed using 1-way ANOVAs followed by pairwise testing with Mann-Whitney *U* tests. Asterisks indicate *P* < 0.05 vs. air-exposed mice belonging to the same genotype or the group indicated.

To determine whether the increased NF-κB activation detected in the lungs of CS-exposed *Cc16^–/–^* mice directly contributes to their exaggerated pulmonary inflammatory response to CS, mice were exposed to CS for 12 weeks and treatment with a small molecule NF-κB inhibitor (IMD0354, IκB kinase-β [IKK2] inhibitor) or vehicle was initiated at the midpoint of these exposures and continued for the second 6 weeks of the exposure (Figure 1D). Treating CS-exposed WT and *Cc16^–/–^* mice with IMD0345 reduced BAL total leukocyte and PMN counts in both genotypes, BAL macrophage counts in *Cc16^–/–^* mice, and BAL lymphocyte counts in WT mice when compared with counts in CS-exposed and vehicle-treated control mice (Figure 10, C–E, and Supplemental Figure 7A). Thus, rhCC16 protein treatment may reduce the exuberant lung inflammatory response to CS in *Cc16^–/–^* mice, in part, by reducing NF-κB activation in their lungs. Although the 12-week CS exposure was insufficient to induce significant airspace enlargement in WT mice, it was sufficient to induce significant SAF in both genotypes (Figure 11). CS-exposed and rhCC16-treated *Cc16^–/–^* mice had significantly lower SAF than CS-exposed and vehicle-treated *Cc16^–/–^* mice (Figure 11, A and B). Thus, excessive activation of NF-κB in the lungs of CS-exposed *Cc16^–/–^* mice is partially responsible for their exaggerated COPD-like disease (27, 28).

## Discussion

**Figure 12:**
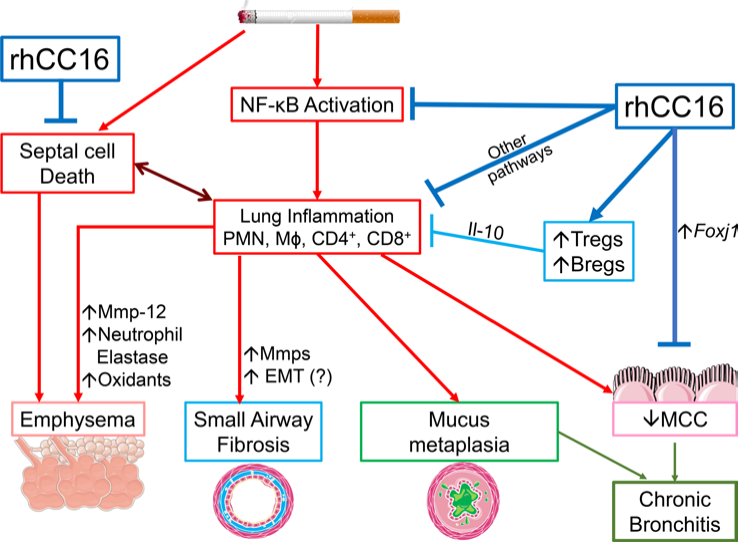
Beneficial effects of rhCC16 on COPD-like disease in CS-exposed mice. Exposing WT and *Cc16^–/–^* mice to CS induces a chronic pulmonary inflammatory response (with increases in PMN, macrophage [Mɸ], and CD4^+^ and CD8^+^ lymphocyte counts) mediated, in part, by increased NF-κB activation in the lungs. These leukocytes (and activated epithelial cells) release metalloproteinases (Mmps), and/or neutrophil elastase (NE), other proteinases, oxidants, and growth factors that promote emphysema development and SAF, and/or increase *Muc5ac* and *Muc5b* expression in airway epithelial cell (mucus metaplasia). CS also promotes emphysema development by increasing alveolar septal cell apoptosis. CS itself and increased levels of PMN-derived NE and Muc5b in CS-exposed airways impair mucociliary clearance (MCC). Epithelial cell mucus metaplasia and impaired MCC contribute to CB-like disease in CS-exposed mice. Delivering rhCC16 to the lungs of CS-exposed mice limits the progression of CS-induced emphysema development, SAF, and CB-like disease in WT and *Cc16^–/–^* mice likely by reducing the pulmonary inflammatory response to CS (in part by reducing the exaggerated NF-κB activation in CS-exposed *Cc16^–/–^* lungs) and by reducing alveolar septal cell apoptosis. Treating mice with rhCC16 also reduces pulmonary inflammation by increasing Treg and Breg accumulation the lungs, which release antiinflammatory Il-10. The reduction in inflammation induced by rhCC16 may reduce septal cell death and loss of parenchymal architecture. In addition, rhCC16 may limit the progression of CB-like disease in mice by improving MCC by reducing airway mucus cell metaplasia and increasing the expression of *Foxj1*, which is required for the generation of motile cilia on epithelial cells. EMT, epithelial-mesenchymal transition.

RhCC16 treatment reduced the progression of COPD-like disease (emphysema development, SAF, and CB) in WT and *Cc16^–/–^* mice (Figure 12). These changes were associated with reductions in processes that contribute to CS-induced COPD-like disease in mice, including: 1) pulmonary inflammation in WT and *Cc16^–/–^* mice, 2) the expression of mediators of inflammation and injury (including Mmps) in the lungs of *Cc16^–/–^* mice, 3) alveolar septal cell apoptosis in WT and *Cc16^–/–^* mice, and 4) NF-κB activation in *Cc16^–/–^* mice. We report for the first time to our knowledge that the increased NF-κB activation associated with the exuberant inflammatory response to CS in *Cc16^–/–^* mice directly contributed to their increased COPD-like disease, as delivering an NF-κB inhibitor to *Cc16^–/–^* mice reduced their exaggerated pulmonary inflammatory response and SAF, and rhCC16 reduced TNF-α–induced NF-κB activation in A549 NF-κB luciferase reporter cells in vitro.

Global Initiative for Chronic Obstructive Lung Disease guidelines propose treating COPD patients with short-acting and/or long-acting inhaled bronchodilators and then adding on inhaled corticosteroids as disease severity increases (1, 29). Several combination inhaled therapies modestly improve forced expiratory volume in 1 second (FEV_1_) but do not substantially improve quality of life or eliminate lung function decline in patients with COPD (30–32). Long-acting β_2_ agonist, long-acting muscarinic antagonist, and corticosteroid triple inhaled combination therapies have reduced mortality in patients with COPD (33, 34) due to reductions in cardiovascular related mortality. Thus, there is an urgent need to develop novel therapies that modify the course of COPD (1).

A prior study reported that treating WT mice with rat rCC16 reduced lung inflammation, emphysema development, and activation of NF-κB in their lungs in a 24-week CS exposure model (35). However, unlike our study, this prior study did not evaluate the effects of rCC16 protein on CS-exposed *Cc16^–/–^* mice, CS-induced SAF, or CB-like disease in WT mice. We tested the efficacy of rhCC16 protein in mice as it is the form that would be advanced to clinical trials in patients with COPD.

### Emphysema.

Lung inflammation (especially increasing in macrophages) is required for CS-induced emphysema development in mice (36, 37). Macrophages contribute to CS-induced emphysema development in mice, in part, by releasing Mmp-12 (which degrades lung elastin) and oxidants (4). PMNs recruited to the lungs initiate and amplify lung inflammation and destruction by releasing proteinases and oxidants (36–38). In acute and/or chronic CS exposures, rhCC16 treatment in WT and *Cc16^–/–^* mice reduced lung NF-κB activation and contributed to reduced macrophage and/or PMN counts, leading to reductions in *Mmp-9* and *Mmp-12* expression levels in the lungs (as these cells are sources of these proteinases) and reductions in emphysema. Macrophages could be a key target cell for rhCC16 given the important contributions of macrophages to CS-induced inflammation and emphysema development and the known activities of CC16 and NF-κB in inhibiting and increasing macrophage activation, respectively (39). Although the CS-exposed lung represents a proteolytic environment, rhCC16 exhibited robust antiinflammatory activities. RhCC16 protein is detectable for several days after delivery to inflamed lungs containing elevated levels of proteinases, including after delivering of a single dose of rhCC16 to animals with experimental acute lung injury or human infants with acute respiratory distress (40–42). We are not aware of any reports identifying proteolytic digestion products of CC16 proteins in biological samples. Thus, rhCC6 may be at least partially resistant to proteolysis in vivo, a feature that is desirable in a protein therapeutic for COPD.

RhCC16 may have reduced lung inflammation and emphysema development in mice via antiinflammatory activities exerted on lung epithelial cells and/or immune cells. Increased NF-κB activation in airway epithelial cells drives pulmonary inflammatory responses to insults other than CS (43, 44). Thus, rhCC16 treatment may have suppressed NF-κB activation in airway epithelial cells to reduce epithelial expression of pro-inflammatory mediators that recruit and activate myeloid leukocytes (45). RhCC16 may also reduce pulmonary inflammation via direct effects on myeloid leukocytes as rhCC16 reduces PMN and monocyte chemotaxis in vitro (46). RhCC16 may also reduce pulmonary inflammation by inhibiting activation of phospholipase A_2_ (which generates pro-inflammatory lipids) by sequestering a cofactor required for phospholipase A_2_ activation (47).

### Tregs and Bregs.

Tregs are antiinflammatory effector cells that are increased or decreased in different lung compartments, or their function is impaired in COPD patients (48). TIGIT expression on Tregs is required for effective suppression of Th1 and Th17 responses and is mediated via TIGIT binding to CD155 expressed on target cells (24). ST2^+^ expression on Tregs is required for Treg accumulation in injured lungs (49). Surprisingly, CS exposure increased total Treg counts and ST2^+^ and TIGIT^+^ Treg counts in *Cc16^–/–^* but not WT mice. The increased Treg accumulation in CS-exposed *Cc16^–/–^* lungs was probably insufficient to limit CS-induced increases in myeloid leukocyte counts and associated injury to the lung. To our knowledge, we report for the first time that the beneficial effects of rhCC16 on the CS-exposed lung may be due, in part, to increased accumulation of activated Tregs, which may restrain exuberant Th1 and Th17 responses to CS.

B cells contribute to lymphoid follicle development in the lungs of human COPD patients and CS-exposed mice and to COPD progression (50–52). B cell subsets have different functions (52). Bregs produce Il-10 and are defined in mice by their expression of Tim-1 (26). While Bregs downregulate adaptive immune responses in other diseases (53), their contributions to COPD are not known. Breg counts were higher in CS-exposed *Cc16^–/–^* mice than WT mice, and treating mice with rhCC16 increased Breg counts in CS-exposed WT and *Cc16^–/–^* mice. Tim-1^+^ B cells induce T cell expression of *FOXP3* and differentiation toward the Treg phenotype (54). Thus, rhCC16 treatment may increase Foxp3^+^ Treg accumulation in CS-exposed lungs by increasing the frequency of pulmonary Bregs.

### Dysfunctional adaptive immunity.

T cell exhaustion, a state of T cell dysfunction, contributes to the poor control of pulmonary infections in COPD patients, and likely promotes infective exacerbations (55). T cell exhaustion is characterized by the surface expression of immune checkpoint molecules including PD-1 and TIM-3 (23). We report for the first time to our knowledge that CS exposure increased pulmonary Tim-3^+^CD4^+^ lymphocyte counts in *Cc16^–/–^* (but not WT) mice and increased pulmonary Tim-3^+^CD8^+^ lymphocyte counts to a greater extent in *Cc16^–/–^* than WT mice. Thus, the reduced CC16 levels that are associated with COPD may contribute to impaired effector T cell responses to pathogens and COPD exacerbations (56). Although rhCC16 treatment did not reduce PD-1 or Tim-3 expression on T or B lymphocytes, or NK cells in CS-exposed WT or *Cc16^–/–^* lungs, future studies should investigate the contributions of CC16 to adaptive immunity and immune tolerance/exhaustion in murine models of COPD exacerbations.

### Septal cell apoptosis.

Excessive alveolar septal cell apoptosis and defective alveolar septal cell repair contribute to emphysema development in CS-exposed animals (57). We report for the first time to our knowledge that rhCC16 treatment reduced CS-induced alveolar septal cell apoptosis in WT and *Cc16^–/–^* mice but had no effect on alveolar septal cell proliferation or activation of the pro-repair Wnt/β catenin pathway in the lungs of either genotype. CC16 treatment may have reduced alveolar septal cell apoptosis either via a direct effect on lung epithelial cells or secondary to its effects on reducing pulmonary inflammation.

### SAF.

SAF contributes to airflow obstruction in COPD and is induced by inflammation and increased expression of growth factors in CS-exposed airways, leading to myofibroblast activation and EMT (58). RhCC16 treatment reduced CS-induced SAF in both genotypes in a Tgf-independent fashion. However, rhCC16 may reduce SAF by other mechanisms not evaluated in this study including by reducing EMT, airway fibroblast activation, and/or epithelial *Muc5b* expression (59).

### CB.

Patients with CB have reduced plasma CC16 levels, and reduced plasma CC16 levels are indirectly related to rate of decline in FEV_1_ in patients with CB (12). However, it has not been clear whether CC16 contributes directly to this COPD phenotype. We report for the first time to our knowledge that CS-exposed *Cc16^–/–^* mice had greater CB-like disease than WT mice as assessed by greater increases in mucin gene expression and greater impairment of MCC. Treating CS-exposed WT and *Cc16^–/–^* mice with rhCC16 improved CB-like disease by reducing CS-induced increases in *Muc5ac* and/or *Mub5b* expression in the lungs and reversing CS-induced impaired MCC in both genotypes. These data support the notion that CC16 deficiency directly contributes to progression of CB in humans and that CC16 augmentation approaches might have utility in reducing progression of this important COPD phenotype.

Mucin gene expression is regulated by several transcription factors, including NF-κB (60), and rhCC16-induced reductions in NF-κB activation in CS-exposed lungs may have contributed to the reduced mucin expression observed in WT and *Cc16^–/–^* mice. Effective MCC requires epithelial cells with beating cilia, which move inhaled particles and pathogens toward the pharynx where they are cleared (16, 17). The airway surface layer contains Muc5ac and Muc5b, and Mub5b is crucial for normal MCC in mice (60, 61). Ciliary injury is caused by CS exposure, which promotes autophagic removal of cilia, neutrophil elastase released by activated airway PMNs, and alterations in mucus rheology (16, 62). *Foxj1* expression by epithelial cells is crucial for the generation of motile cilia (17, 18). The improvement in MCC mediated by rhCC16 treatment of CS-exposed WT and *Cc16^–/–^* mice was likely mediated by rhCC16-induced increases in *Foxj1* expression, and reductions in mucin gene expression and pulmonary PMN counts leading to reduced lung levels of neutrophil elastase.

### NF-κB pathway.

NF-κB activation occurs in the lungs of COPD patients and may contribute to the pathogenesis of COPD (10), but this hypothesis has not been proved. The exaggerated COPD-like disease in CS-exposed *Cc16^–/–^* mice is associated with increased NF-κB activation in their lungs (10). Treating CS-exposed WT mice with rat CC16 reduced activation of NF-κB in their lungs (35). In our study, treating CS-exposed *Cc16^–/–^* (but not WT) mice with rhCC16 was associated with reduced NF-κB activation in their lungs. Our study supports that the increased NF-κB activation observed in CS-exposed *Cc16^–/–^* lungs directly contributes to their exaggerated pulmonary inflammation and SAF as both lesions were reduced by treating CS-exposed *Cc16^–/–^* mice with a small molecule (IKK2) NF-κB inhibitor. This notion is further supported by the finding that rhCC16 reduced NF-κB activation in an NF-κB luciferase reporter cell line activated with TNF-α (a cytokine that is elevated in blood samples from COPD patients; ref. 63). The NF-κB inhibitor did not completely inhibit CS-induced pulmonary inflammation in *Cc16^–/–^* (or WT) mice, and rhCC16 had a greater impact on several lung pathologies than the NF-κB inhibitor, indicating that rhCC16 mediates its beneficial activities in the CS-exposed lung on signaling pathways other than NF-κB. NF-κB is regulated by different mechanisms, and it is possible that the NF-κB inhibitor’s inhibitory activities were different from those mediated by rhCC16. IKK2 regulates other pathways (64), including cell cycle control and mTOR activation, which were not evaluated in this study.

### Limitations.

Our study has limitations in the mechanistic approaches. Although CC16 mediates its protective activities in the CS-exposed lung, in part, by inhibiting NF-κB activation, it likely has beneficial activities on other pathways, which we did not identify. We did not determine whether rhCC16 acts on epithelial cells or immune cells or both. This would require the generation of tagged rhCC16, which would be technically challenging given the small size of CC16 (16 kDa) relative to that of standard fluorescent tags (e.g., green fluorescent protein [GFP; M_r_ ~26 kDa], which has a secondary β-barrel structure). GFP tagging would likely impede the binding of CC16 to its effector molecule via steric hindrance. Newer, smaller tags using only portions of fluorescent tags (65) fused with rhCC16 could be used in future studies to identify the cells to which rhCC16 binds.

We also did not identify the receptor(s) or extracellular molecules to which CC16 binds to mediate its protective effects. CC16 binds to VLA4 integrin and the lipoxin A4 receptor on leukocytes to reduce their activation (66, 67) and the lipocalin-1 receptor on cancer cells to suppress their invasiveness (68). TRICEPS technology (69) will allow the identification of cell surface receptors to which CC16 binds. Future studies will determine whether epithelial and/or immune cells are the key target cells for CC16, the target receptor(s) to which rhCC16 binds, and the genes that are regulated by rhCC16 in these cells.

We did not determine whether rhCC16 restores MCC in CS-exposed mice by promoting ciliogenesis and/or ciliary repair. These mechanistic approaches are beyond the scope of this manuscript but will be the focus of future studies. We were unable to measure lung volumes (as we lack the necessary equipment) or to develop an assay to measure anti-CC16 antibodies in plasma samples in rhCC16-treated mice. Although we demonstrated differences between the *Cc16^–/–^* groups, the experiments were not powered to show differences in the WT groups (the power was 0.223 for the WT groups). A sample size calculation indicated that 26–33 WT mice per group would be needed to achieve statistical significance (with power of 0.8), which is twice the sample size recommended in ARRIVE guidelines (70). Finally, inflammatory mediator expression was measured in whole lung samples, which does not provide information on their cellular sources.

### Conclusions.

Delivering rhCC16 to the lung limited the progression of CS-induced emphysema, SAF, and CB-like disease in WT and *Cc16^–/–^* mice, in part, by reducing the pulmonary inflammatory response to CS. Treating *Cc16^–/–^* mice with rhCC16 protein reduced their exuberant inflammatory response to CS, in part, by reducing NF-κB activation in their lungs. RhCC16 treatment also reduced CS-induced COPD-like disease in both genotypes via effects on lung epithelial cells by reducing alveolar septal cell apoptosis (thereby reducing emphysema development) and reducing airway mucus cell metaplasia and improving MCC (thereby reducing CB-like disease). We also report potentially novel activities for rhCC16 treatment in reducing adaptive immune responses to CS by increasing functional Treg and Breg cell counts in the CS-exposed lung. These results provide a rationale for evaluating the safety, tolerability, and efficacy of rhCC16 protein augmentation therapy in clinical trials of patients with COPD.

## Methods

See Supplemental Methods for additional details.

### Animals.

*Cc16^–/–^* mice were bred in C57BL/6 background and have no abnormalities in the unchallenged state (71). C57BL/6 WT mice were obtained from the Jackson Laboratory. *Cc16^–/–^* and WT mouse colonies were housed under identical specific pathogen–free conditions in a barrier facility. *Cc16^–/–^* genotype was confirmed using PCR-based protocols performed on DNA extracted from tail biopsies. Adult male and female WT and *Cc16^–/–^* mice were used in all experiments and randomized to experimental groups by an individual who was not involved in the conduct of the experiments.

### CS exposures.

WT and *Cc16^–/–^* mice were exposed to air or mixed mainstream and side-stream CS from 1R3F Kentucky Research (University of Kentucky, Lexington, Kentucky, USA) cigarettes 6 days per week in Teague chambers (Figure 1).

### RhCC16 protein.

This protein (provided by APCBio Innovations, Inc.) was expressed in *E*. *coli* and isolated as a 16.1 kDa disulfide-linked homodimer. Purity was assessed using SDS-PAGE and the inhibitory activity of rhCC16 against soluble phospholipase A_2_ (Cayman Chemical) was measured as described previously (72). All preparations produced ≥90% inhibition of soluble phospholipase A_2_ activity.

### RhCC16 treatments.

The initial dose and route of administration for rhCC16 were selected based on previous studies (41, 42). However, we conducted a pilot study in mice by administering rhCC16 (75 μg diluted in 30 μL of endotoxin-free PBS [~2.1–3.75 mg/kg]) or vehicle [endotoxin-free PBS]) thrice weekly during the second 4 weeks of 8-week CS exposures to optimize the dose and dosing frequency before conducting chronic CS exposures in mice. The 75 μg thrice weekly dose of rhCC16 substantially reduced CS-induced acute pulmonary inflammation (Figure 5). Thus, the same dose and thrice weekly dosing regimen was used in all subsequent CS exposure experiments. Mice were exposed to air or CS for 5 or 8 weeks (acute exposures), or 24 weeks (chronic exposures), and CS-exposed mice were treated with rhCC16 or vehicle thrice weekly for the last half of the exposures (Figure 1). Mice were anesthetized with inhaled isoflurane, and rhCC16 or PBS was delivered via the i.n. route 45 minutes after the CS exposure ended.

### NF-κB inhibitor studies.

Mice were exposed to air or CS for 12 weeks, and CS-exposed mice were treated with IMD0354 (an IKK2 inhibitor; Bio-Techne) at a dose of 6 mg/kg in 200 μL of vehicle (endotoxin-free 0.9% saline solution containing 0.5% ethanol and 1% Tween 80) or 200 μL of vehicle alone delivered by the intraperitoneal route once daily beginning at the midpoint of the exposures. The IMD0354 dose selected was based on data from studies showing that this dose abrogated ischemia/reperfusion injury in rats (73). At the end of the exposures, mice were euthanized, BAL was performed, and lungs were inflated and fixed in formalin (19).

### Emphysema and SAF.

Inflated lung sections (14) were stained with Gill’s to quantify airspace enlargement. Briefly, lung sections were stained with a mixture of equal parts of Gill’s no. 3 and Harris modified hematoxylin solution (both MilliporeSigma). Images of both lungs were captured at 200× original magnification using a Nikon Eclipse Ts2 microscope (15–20 images/mouse). Scion Image software (Scion Corp.) was used to measure alveolar chord lengths (14, 19). SAF was measured on Masson’s trichrome–stained lung sections. Briefly, the mean airway luminal diameter and the thickness of the subepithelial layer of extracellular matrix protein deposited around airways having a mean diameter of 300–699 microns were measured at 12 equidistant sites around each airway using MetaMorph software (Molecular Devices). The mean ± SEM thickness of the subepithelial layer of extracellular matrix layer in microns was calculated. Sections of airways sharing their adventitia with arteries were not included in the analysis (14, 19).

### MCC.

Mice were exposed to CS for 5 weeks. Mice were treated thrice weekly with rhCC16 or vehicle during the last 2 weeks of the 5-week CS exposures. ^99m^Tc-sc was instilled into the lungs of anesthetized mice by oropharyngeal aspiration, and the efficiency of ^99m^Tc-sc clearance from lungs was measured with noninvasive 3D μ-SPECT functional imaging, as described previously (16).

### Pulmonary inflammation.

Total leukocytes and leukocyte subsets were counted in BAL samples (19). Right lungs were enzymatically digested (20), and single-cell suspensions were stained for markers of T or B lymphocytes, or NK cells, or lymphocyte effector cell function or exhaustion (Supplemental Table 4) and analyzed using flow cytometry.

### Respiratory mechanics.

After the last day of CS or air exposure, mice were anesthetized, a tracheostomy was performed, and an 18-gauge cannula was inserted in the trachea (14). The animals were connected by the cannula to a digitally controlled mechanical ventilator (FlexiVent device; Scireq Inc.). Respiratory mechanics were measured, and then the mice were humanely euthanized.

### Gene expression studies.

The expression of *Ccl-2*, *Ccl-3*, *Ccl-5*, *Il-6*, *Tnf-*α, *Il-10*, *Tgf-β*, *Foxj1*, *Muc5ac*, *Muc5b*, *Muc1*, *Mmp-9*, *Mmp-12*, *p16*, *p21*, *Sirtuin*, *Wisp*, and *Tcf-7* was quantified in lungs using RT-qPCR, *Ppia* as the housekeeping gene, and the comparative threshold method. Supplemental Table 5 shows the primer sequences used.

### Immunofluorescence staining of lung sections.

Lung sections were immunostained for Foxj1, Muc5ac, cleaved caspase-3, or Ki67, or with appropriate nonimmune IgGs (Thermo Fisher Scientific), as described in the Supplemental Methods.

### NF-κB activation.

Proteins were isolated from nuclear extracts of whole lungs using a kit (Abcam). NF-κB was quantified in nuclear protein extracts using a TransAM NF-κB kit (Active Motif).

### NF-κB luciferase assay.

NF-κB luciferase reporter A549 cells (Lonza) (74) were preincubated with 100 μg of rhCC16 for 3 hours, followed by either CSE at 1%–10% or 1–10 ng/mL rhTNF-α (Peprotech). See Supplemental Methods for more details.

### Statistics.

Statistical analyses were performed using SigmaPlot software (Systat). Sample sizes were calculated based on an estimated effect size of a 20% difference between the groups, 80% power, and α = 0.05 resulting in planned samples sizes of 4–10 mice per group. The Shapiro-Wilk test was used to determine normality, and the Brown-Forsythe test was used to assess equal variance. Data were analyzed using 1-way ANOVAs for continuous data, followed by post hoc testing with 2-sided Student’s *t* tests or Mann-Whitney *U* test, according to distribution. Parametric data are presented as mean ± SEM, and nonparametric are presented as box plots showing medians and 25th and 75th percentiles and whiskers showing 10th and 90th percentiles. *P* < 0.05 was considered statistically significant.

### Study approval.

All procedures performed on mice were approved by the Brigham and Women’s Hospital (BWH) Institutional Animal Care and Use Committee.

## Author contributions

CAO designed the project. CAO, JRQ, MELC, and ALP designed the experiments. CAO, JRQ, MELC, XW, QAF, PRB, SJK, DZ, YL, LZ, and HK conducted experiments and analyzed data. JRQ, MELC, ARB, FP, YT, BC, ALP, and CAO contributed to data interpretation. JRQ and CAO wrote the manuscript. All authors reviewed and approved the manuscript.

## Supplementary Material

Supplemental data

## Figures and Tables

**Table 1:**
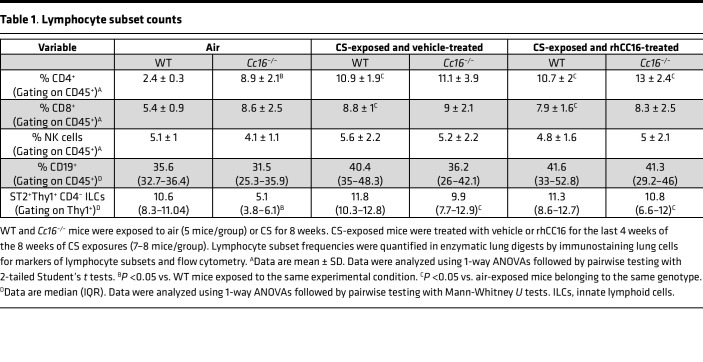
Lymphocyte subset counts

## Version 1 02/14/2023

In-Press Preview

## Version 2 03/22/2023

Electronic publication
