# Supplementary material for: CC16 augmentation reduces exaggerated COPD-like disease in Cc16-deficient mice
Source: JCI Insight. 2023 Mar 22;8(6):e130771. doi: 10.1172/jci.insight.130771 (PMC10070105; doi:10.1172/jci.insight.130771)
Supplement: Supplemental data [file jciinsight-8-130771-s200.pdf]

ONLINE SUPPLEMENT

MATERIALS AND METHODS

Animal experiments: Procedures performed on mice were approved by the Brigham and Women's Hospital Institutional Animal Care and Use Committee. *Cc16*$^{-/-}$ mice in a pure C57BL/6 background (1) have a normal lifespan and fertility, and have no abnormalities in major organs in the unchallenged state (1). Colonies of *Cc16*$^{-/-}$ mice and C57BL/6 wild-type (WT) mice were housed under identical specific pathogen-free conditions in a barrier facility. The genotype of the *Cc16*$^{-/-}$ mice was confirmed using PCR-based protocols performed on genomic DNA extracted from tail biopsies. Adult male and female WT and *Cc16*$^{-/-}$ mice were used in all experiments and were randomized to experimental groups by an individual who was not involved in the conduct of the experiments.

Cigarette smoke (CS) exposures: Male and female WT and *Cc16*$^{-/-}$ mice were exposed to air or mixed mainstream and side-stream CS from 1R3F Kentucky Research cigarettes 6 days/week in Teague chambers for 3-24 weeks beginning at 8-10 weeks of age (3). The CS exposures produced daily total suspended particles counts ~150 mg/m^3. Mice were weighed at baseline and every two weeks thereafter.

Treatment of mice with rhCC16 protein solution: The initial dose and route of administration for rhCC16 were selected based on previous studies (4, 5). However, we conducted a pilot study in mice by administering rhCC16 (75 µg diluted in 30 µL of endotoxin-free PBS [~2.1-3.75 mg/kg]) or vehicle [endotoxin-free PBS]) thrice weekly during the second 4 weeks of 8-week CS exposures to optimize the dose and dosing frequency before conducting chronic CS exposures in mice. PBS rather than heat inactivated rhCC16 was used as the control as rhCC16 retains activity (assessed as PLA_2 inhibitory activity) after it is heated for 1 h at 95°C (ALP unpublished observation). The 75 µg thrice weekly dose of rhCC16 substantially reduced in CS-induced pulmonary inflammation. Thus, thereafter the same dose and thrice weekly dosing regimen was used in all CS exposure experiments. Mice were exposed to air or CS for 5 or 8 weeks (acute exposures), or 24 weeks (chronic exposures), and CS-exposed mice were treated with rhCC16 protein solution or vehicle thrice weekly beginning at the mid-point of the exposures and continuing throughout the second half of the exposures. To deliver the rhCC16 or vehicle, mice were anesthetized with inhaled isoflurane, and rhCC16 protein solution or PBS were delivered via the i.n. route 45 min after the CS exposure ended.

Acute CS exposures and rhCC16 treatments: WT and *Cc16*$^{-/-}$ mice were exposed to CS for 4 weeks (to induce an acute pulmonary inflammatory response in the lungs). CS-exposed mice were then treated thrice weekly with rhCC16 protein solution or vehicle during an additional 4 weeks of CS exposures (8 weeks of CS exposure in total). Control mice were exposed to air for 8 weeks. At the end of the exposures, mice were euthanized, bronchoalveolar lavage (BAL) was performed, BAL leukocytes were counted and lungs were removed and stored at 80°C for analysis.

Chronic CS exposures and rhCC16 treatments: WT and *Cc16*$^{-/-}$ mice were exposed to CS for 12 weeks (to initiate a chronic inflammatory response and emphysema development). CS-exposed mice were then treated thrice weekly with rhCC16 protein solution or vehicle during an additional 12 weeks of CS exposures (24 weeks of CS exposure in total). Control mice were exposed to air for 24 weeks. At the end of the experiment, respiratory mechanics were measured as outlined below. Mice were then euthanized, and BAL was performed and then lungs were inflated to 25 cm H_2O pressure and fixed for 24 h with 10% saline-buffered formalin (3). Lung sections were used to quantify emphysema development, small airway fibrosis, and airway epithelial cell mucus metaplasia, as outlined below.

NF -κB activation Assay: Proteins were isolated from nuclear extracts of whole lungs using a kit (Abcam, Cambridge, MA). NF-κB was quantified in nuclear protein extracts using a TransAM® NF-κB kit (Active Motif, Carlsbad, CA) in which NF-κB protein that has translocated to the nucleus binds to an

oligonucleotide containing the NF-κB consensus-binding site sequence immobilized to 96-well plates and is detected using an antibody to the p65 NF-κB subunit.

NF-κB inhibitor studies: WT and *Cc16*$^{-/-}$ mice were exposed to air or CS for 12 weeks. CS-exposed mice were treated with IMD-0354 (N-[3,5-Bis(trifluoromethyl)phenyl]-5-chloro-2-hydroxybenzamide; an IκB Kinase-β inhibitor, which inhibits NF-κB translocation to the nucleus) beginning at the mid-point of the exposures. IMD-0354 (6 mg/kg body weight in 100 µL of vehicle) or the same volume of vehicle (0.9% saline containing 0.5% ethanol and 1% Tween-80) delivered daily by the intraperitoneal route. The IMD0354 dose evaluated was selected based on the results of studies showing that this dose abrogated ischemia-reperfusion injury in rats (6). At the end of the exposures, mice were euthanized, BAL was performed, and BAL total leukocyte and leukocyte subsets were counted, as described below. The lungs were then inflated and fixed in formalin as described above, and small airway remodeling was quantified as described below.

Bronchoalveolar lavage (BAL): Absolute numbers of all leukocytes and leukocyte subsets were counted in BAL samples from air- and CS-exposed mice (2). Briefly, BAL was performed on euthanized mice using 8 X 0.5 mL aliquots of PBS (2). BAL leukocytes were counted, and cyto-centrifuge preparations were stained with modified Wright's stain, differential leukocyte counts were performed, and absolute numbers of leukocyte subsets were calculated.

Airspace enlargement: Mid-sagittal sections of lungs (8 µm thick) were stained with Gill's stain. The images of both lungs were captured at 200 X magnification using a Nikon microscope (15-20 images per mouse). Scion Image software (Scion Corp., Frederick, MD, USA) was used to measure alveolar chord lengths, as described previously (3, 7).

Respiratory mechanics: After the last CS or air exposure, mice were anesthetized with a cocktail of 100 mg/kg ketamine, 10 mg/kg xylazine, and 3 mg/kg acepromazine. A tracheostomy was performed, and an 18-gauge cannula was inserted and secured in the trachea using sutures. The animals were connected by the cannula to a digitally-controlled mechanical ventilator (Flexi Vent device; Scireq Inc., Montreal, QC, Canada). Ventilator settings were f = 150/min, FiO_2 = 0.21, tidal volume =10 ml/kg body weight, and positive end-expiratory pressure (PEEP) of 3 cm H_20. Murine lungs were inflated to total lung capacity (TLC; 25 cm H_2O) 3 times for volume history. Tissue resistance (G) and tissue elastance (H) at PEEP of 3 cm H_2O were then measured, followed by dynamic compliance, stepwise quasi-static compliance (Cst), and volume-pressure curves. Mice were humanely euthanized at the end of the measurements, and the lungs were inflated and removed (3, 7).

Small airway fibrosis: Lung sections from mice were stained with Masson's Trichrome stain, and images of both lungs were acquired at 200 X magnification. The mean airway luminal diameter and the thickness of the sub-epithelial layer of extracellular matrix protein deposited around airways having a mean diameter of 300-699 microns were measured at 12 equidistant sites around each airway using MetaMorph® software (Molecular Devices, LLC, 2018). The mean ± SEM thickness of the subepithelial layer of extracellular matrix layer in microns was calculated for airways of. Sections of airways sharing their adventitia with arteries were not included in the analysis (3, 7).

Mucociliary clearance (MCC): MCC is completely lost when WT mice are exposed to CS for 3 weeks (8). WT and *Cc16*$^{-/-}$ mice were exposed to air or CS for 3 weeks. Other cohorts of mice were exposed to CS for 5 weeks and treated with rhCC16 protein solution or vehicle for the last 2 weeks of the 5 week CS exposures. At the end of the exposures, mice were anesthetized with a cocktail of 100 mg/kg ketamine and 10 mg/kg xylazine, and 0.3-0.5 mCi of ^{99m}Technetium-sulfur colloid particles (^{99m}Tc-sc) suspended in 50 µL of PBS was delivered to each mouse using the oropharyngeal aspiration method. The efficiency of ^{99m}Tc-sc clearance from the lungs was measured using non-invasive functional imaging (three dimensional whole-mouse µ-Single Photon Emission Computed Tomography imaging (3D µ-SPECT) (9) with a TRIAD XLT-20 triple head-detector SPECT system (Triad XLT, Trionix Research Laboratories, Twinsburg, OH).

The 3D images were reconstructed using an ordered-subsets expectation-maximization algorithm (10), and consisted of 80 x 80 (transaxial) x 200 (axial) isotropic voxels each having 0.4 x 0.4 x 0.4 mm^3 dimensions. The reconstructed image volumes taken at baseline, and 1 h and 3 h later were analyzed using ImageJ (https://imagej.nih.gov/ij/), creating a new image from "sum slices" z-projection over 80 coronal images. The regions of interest were drawn to exclude the trachea and gastrointestinal tract. Retention at each time point was subtracted from 1.00 and multiplied by 100% to obtain the percent mucociliary clearance (9, 10).

Lymphocyte subsets in enzymatic lung digests: WT and *Cc16$^{-/-}$* mice were exposed to CS for 4 weeks to induce an acute pulmonary inflammatory response in the lungs or to air for 8 weeks as a control. CS-exposed mice were treated thrice weekly with rhCC16 protein solution or vehicle during an additional 4 weeks of CS exposures (8 weeks of CS exposure in total). The right lungs were digested in a 1 mg/mL collagenase D (Sigma Aldrich, St. Louis, MO) solution at 37°C for 30 min, as described previously (11). Samples were passed through a 70 µm strainer and the resulting single-cell suspension was washed twice with cold PBS. Cell viability was quantified with trypan blue dye, and was >90% in all experiments. Surface staining for markers of leukocytes and leukocyte subsets was performed for 20 min at 4°C in PBS containing 2% fetal calf serum. Intracellular staining for Foxp3 was performed using the Foxp3 Staining Buffer Set (eBioscience, San Diego, CA). T and B lymphocyte panels and antibodies used are shown in **Table E4**. Flow cytometry was performed using a BD LSRFortessa Cell Analyzer (San Jose, CA) and analyzed using FlowJo software (Tree Star, Inc., Ashland, OR).

Quantification of the expression of mediators of inflammation, mucins, senescence markers, and activation of the Wnt-β catenin pathway in whole lung samples: WT and *Cc16$^{-/-}$* mice were exposed to CS for 4 weeks (to induce an acute pulmonary inflammatory response in the lungs) or to air for 8 weeks as a control. CS-exposed mice were treated thrice weekly with rhCC16 protein solution or vehicle during an additional 4 weeks of CS exposures (8 weeks of CS exposure in total). RNA was extracted from murine lungs using a SurePrep TrueTotal RNA Purification Kit (Fisher Scientific, Fair Lawn, NJ). RNA (1 µg) was reverse transcribed into cDNA using a High-Capacity cDNA Reverse Transcription Kit (ThermoFisher Scientific, Carlsbad, CA). Real-time RT-PCR was performed to quantify the expression of *Ccl-2*, *Ccl-3*, *Ccl-5*, *Il-6*, *Tnf-α*, *Il-10*, *Tgf-β*, *Foxj1*, Muc*5ac*, Muc*5b,* Muc*1*, *Mmp-9*, *Mmp-12*, *p16*, *p21*, *Sirtuin*, *Wisp*, and *Tcf-7*, using *Ppia* as the housekeeping gene (see **Table E5** for primer sequences), and an AriaMx Real time PCR device (Agilent technologies, Santa Clara, CA). Primers and SYBR green dye were obtained from ThermoFisher Scientific (Waltham, MA). The results were expressed as fold change relative to expression of the gene of interest in samples from mice exposed to air.

Alveolar septal cell apoptosis: Sections of lungs from mice were immunostained for active caspase-3. Lung sections were deparaffinized, and antigen retrieval was performed by heating the slides in a microwave in 10 mM citrate buffer (pH 6.0) for 10 min. The sections were blocked overnight at 4°C in PBS containing 1% albumin and 5% normal goat serum. The sections were incubated at 4°C overnight rabbit anti-murine cleaved caspase-3 IgG at a 1:50 dilution (Cell Signaling, Danvers, MA) or non-immune rabbit IgG (Agilent, Carpinteria, CA) applied at the same concentration. After washing the lung sections with PBS, the sections were incubated for 1 h at 37°C with goat anti-murine $F(ab')_2$-conjugated to Alexa 488 (Invitrogen, Carlsbad, CA). After washing the slides twice with PBS, the sections were incubated with Sudan Black buffer for 25 min at room temperature. After washing the slides twice with PBS, nuclei were counterstained with DAPI mounting gel (Abcam, Cambridge, MA). Images were captured using a digital camera, and the number of active-caspase-3-positive cells was quantified. Six 200 X magnification images per mouse were randomly acquired using a Leica microscope and a digital camera. Images were processed using ImageJ software (https://imagej.nih.gov/ij/). Images of the DAPI (stained blue) and active caspase-3-positively-stained cells (stained green) were merged, and active caspase-3 positively-stained alveolar septal cells were counted in ~5,000 alveolar septal cells per mouse (identified by their DAPI-stained nuclei). The results were expressed as % of alveolar septal cells that were positively-stained for active caspase-3.

Alveolar septal cell proliferation: Lung sections from WT and *Cc16*$^{-/-}$ mice were immunostained for Ki-67, a marker of cellular proliferation. Lung sections were deparaffinized, and antigen retrieval was performed by heating the slides in a microwave in 10 mM citrate buffer (pH 6.0) for 10 min. The sections were blocked overnight at 4°C in PBS containing 1% albumin and 5% normal goat serum. After washing the slides with PBS, a rabbit polyclonal anti-murine Ki67 IgG (Abcam, Cambridge, MA) was added to the slides at a 1:100 dilution and incubated overnight at 4°C. A non-immune rabbit IgG (Invitrogen, Carlsbad, CA) was applied to other slides at the same concentration as the immune antibody. After washing the slides twice in PBS, goat anti-murine $F(ab')_2$-conjugated to Alexa 488 (Invitrogen, Carlsbad, CA) at 1:100 dilution was added to the slides, and the slides were incubated at 37°C for 1 h. After washing the slides twice with PBS, the sections were incubated with Sudan Black buffer for 25 min at room temperature. After washing the slides twice with PBS, nuclei were counterstained with DAPI mounting gel (Abcam, Cambridge, MA). Six 200 X magnification images per mouse were randomly acquired using a Leica microscope and a digital camera. Images were processed using ImageJ software (https://imagej.nih.gov/ij/). Images of the DAPI (stained blue) and Ki-67 positively-stained cells (stained green) were merged, and Ki67 positively-stained alveolar septal cells were counted in ~ 5,000 alveolar septal cells per mouse identified by their DAPI-stained nuclei. The results were expressed as % of alveolar septal cells that were positively-stained for Ki-67.

Airway epithelial Muc5ac staining: To identify the Muc5ac-positive cells in bronchial epithelial cells, sections of lung from WT and *Cc16*$^{-/-}$ mice were immunostained for Muc5ac. Lung sections were deparaffinized, and antigen retrieval was performed by heating the slides in a microwave in 10 mM citrate buffer (pH 6.0) for 10 min. The sections were blocked overnight at 4°C in PBS containing 1% albumin and 5% normal rabbit serum. The sections were incubated at 4°C overnight with a murine IgG to Muc5ac (diluted 1:50, ThermoFisher Scientific, Waltham, MA), and non-immune murine IgG applied at the same concentration. After washing the lung sections with PBS, the sections were incubated for 1 h at 37°C with F(ab')2-rabbit anti-mouse conjugated with Alexa 488 (Invitrogen, Carlsbad, CA) for Muc5ac. After washing the slides twice with PBS, the sections were incubated with Sudan Black buffer for 25 min at room temperature. After washing the slides twice with PBS, nuclei were counterstained with DAPI (4′,6-diamidino-2-phenylindol) mounting gel (Abcam, Cambridge, MA). Images were captured using a digital camera, the number of Muc5ac-positive cells was counted and normalized for the bronchial epithelial cell areas using MetaMorph software (Molecular Devices, San Jose, CA).

Foxj1 staining: To identify the Foxj1-positive cells in bronchial epithelial cells, sections of lung from WT and *Cc16*$^{-/-}$ mice were immunostained for Foxj1. Lung sections were deparaffinized, and antigen retrieval was performed by heating the slides in a microwave in 10 mM citrate buffer (pH 6.0) for 10 min. The sections were blocked overnight at 4°C in PBS containing 1% albumin and 5% normal goat serum. The sections were incubated at 4°C overnight with a murine IgG to Foxj1 (diluted 1:200, ThermoFisher Scientific, Waltham, MA), and non-immune murine IgG was applied at the same concentration. After washing the lung sections with PBS, the sections were incubated for 1 h at 37°C with $F(ab')_2$-goat anti-mouse conjugated with Alexa 488 (Invitrogen, Carlsbad, CA). After washing the slides twice with PBS, the sections were incubated with Sudan Black buffer for 25 min at room temperature. After washing the slides twice with PBS, nuclei were counterstained with DAPI (4′,6-diamidino-2-phenylindol) mounting gel (Abcam, Cambridge, MA). Images were captured using a digital camera, the number of Foxj1-positive cells was counted and normalized for the bronchial epithelial cell areas using MetaMorph software (Molecular Devices, San Jose, CA).

Cigarette Smoke Extract (CSE): CSE was prepared as previously described (3). Briefly, CSE was prepared by allowing smoke from two 1R3F Kentucky Research (University of Kentucky, Lexington, KY) to bubble through 20 mL of DMEM medium to yield a 100% CSE solution. CSE was filtered with a 0.2 μm syringe filter (ThermoFisher Scientific, Waltham, MA) and immediately added to cell cultures to yield the stated final concentration of CSE for each experiment (1%, 2,5%, 5%, and 10%).

NF-κB luciferase reporter A549 cells: NF-κB luciferase reporter A549 cells (12) (ATCC, Manassas, VA, USA) were cultured in Costar® 12-well Clear TC-treated Multiple Wells (Washington, DC) until ~80% confluence in Dulbecco`s Modified Eagle Media (Corning, Corning, NY) supplemented with 10% fetal bovine serum (ThermoFisher Scientific, Waltham, MA), penicillin/streptomycin (ThermoFisher Scientific, Waltham, MA) and amphotericin B (ThermoFisher Scientific, Waltham, MA). The cells were pre-incubated with 100 μg of rhCC16 for 3 h. Cells were then incubated with either 1%, 2,5%, 5%, or 10% CSE (final concentrations), or human recombinant TNF-α (Peprotech, Rocky Hill, NJ) at 1-10 ng/mL for 8-24 h. At the end of the experiment, cells were lysed with Promega® 1X Luciferase Cell Culture Lysis Reagent (Madison, WI). Cell lysate (20 µL) was mixed with 100 µL of Luciferase assay working solution according to manufacturer's instruction (Promega, Madison, WI), and luminosity was quantified with a Synergy™HT plate reader and Gen5® software. Experiments were repeated 3 or 4 times.

Statistical Analyses: Statistical analysis was performed using SigmaPlot™ software (Systat, San Jose, CA). The Shapiro-Wilk test was used to determine whether the data were normally distributed, and Brown-Forsythe test was used to assess equal variance. Data were analyzed using one-way ANOVAs for continuous data, followed by post-hoc testing with 2-sided Student's t-tests (normally-distributed data) or Mann-Whitney U tests (not normally-distributed data). Normally-distributed data are presented as mean ± SEM, and not normally-distributed data are presented as box plots showing medians and 25th and 75th percentiles and error bars showing 10th and 90th percentiles. ANOVA for repetitive measures was used to analyze changes in body weight from baseline values. $P < 0.05$ was considered statistically significant.

REFERENCES

1. Stripp BR, Lund J, Mango GW, Doyen KC, Johnston C, Hultenby K, Nord M, Whitsett JA. Clara cell secretory protein: a determinant of PCB bioaccumulation in mammals. *Am J Physiol*. 1996;271(4 Pt 1):L656-64.
2. Wang X, et al. A Disintegrin and A Metalloproteinase-9 (ADAM9): A Novel Proteinase Culprit with Multifarious Contributions to COPD. *Am J Respir Crit Care Med*. 2018;198(12): 1500–1518.
3. Laucho-Contreras ME, et al. Protective role for club cell secretory protein-16 (CC16) in the development of COPD. *Eur Respir J.* 2015;45(6):1544-56.
4. Chandra S, Davis JM, Drexler S, Kowalewska J, Chester D, Koo HC, Pollack S, Welch R, Pilon A, Levine CR. Safety and efficacy of intratracheal recombinant human Clara cell protein in a newborn piglet model of acute lung injury. *Pediatr Res*. 2003;54(4):509-15.
5. Levine CR, et al. The safety, pharmacokinetics, and anti-inflammatory effects of intratracheal recombinant human Clara cell protein in premature infants with respiratory distress syndrome. *Pediatr Res.* 2005 Jul;58(1):15-21.
6. Onai Y, et al. Inhibition of IkappaB phosphorylation in cardiomyocytes attenuates myocardial ischemia/reperfusion injury. *Cardiovasc. Res.* 2004;63(1):51-9.
7. Laucho-Contreras ME, Taylor KL, Mahadeva R, Boukedes SS, Owen CA. Automated measurement of pulmonary emphysema and small airway remodeling in cigarette smoke-exposed mice. J Vis Exp. 2015;(95):52236.
8. Lam HC, Cloonan SM, Bhashyam AR, Haspel JA, Singh A, Sathirapongsasuti JF, Cervo M, Yao H, Chung AL, Mizumura K, An CH, Shan B, Franks JM, Haley KJ, Owen CA, Tesfaigzi Y, Washko GR, Quackenbush J, Silverman EK, Rahman I, Kim HP, Mahmood A, Biswal SS, Ryter SW, Choi AM. Histone deacetylase 6-mediated selective autophagy regulates COPD-associated cilia dysfunction. *J Clin Invest*. 2013;123(12):5212-30.
9. Hudson HM1, Larkin RS. Accelerated image reconstruction using ordered subsets of projection data. *IEEE Trans Med Imaging.* 1994;13(4):601-9.
10. Bhashyam AR, Mogayzel PJ Jr, Cleary JC, Undem BJ, Kollarik M, Fox J, Laube BL. Vagal control of mucociliary clearance in murine lungs: a study in a chronic preparation. *Auton Neurosci*. 2010;154(1-2):74-8.
11. Rojas-Quintero J, Wang X, Tipper J, Burkett PR, Zuñiga J, Ashtekar AR, Polverino F, Rout A, Yambayev I, Hernández C, Jimenez L, Ramírez G, Harrod KS, Owen CA. Matrix metalloproteinase-9 deficiency protects mice from severe influenza A viral infection. *JCI Insight.* 2018;3(24):pii 99022.
12. Marcos-Vadillo E, García-Sánchez A. Promoter Assay Using Luciferase Reporter Gene in the A549 Cell Line. Methods Mol Biol. 2016;1434:199-211.

Table E1. Gender differences in COPD-like disease features in cigarette smoke-exposed WT and *Cc16*$^{-/-}$ mice treated with rhCC16 protein solution.

	**Sex**	**Airspace enlargement [mean ± SD]**[§]	***P******	**Sex**	**Small Airway Remodeling [mean ± SD]**[‡]	***P******
**WT Air**	Male (n = 3)	17.63 ± 0.5	0.37	Male (n = 3)	16.88 ± 1.7	0.73
	Females (n = 3)	17.94 ± 0.2		Females (n = 2)	17.49 ± 2.0	
***Cc16*$^{-/-}$ Air**	Male (n = 3)	18.86 ± 0.5	0.26	Male (n = 3)	22.45 ± 3.9	0.40
	Females (n = 2)	18.31 ± 0.04		Females (n = 2)	22.66 ± 2.1	
**WT 24 wks CS 12 wks Vehicle**	Male (n = 4)	19.07 ± 1.3	0.26	Male (n = 4)	25.46 ± 2.6	0.59
	Females (n = 2)	20.4 ± 0.6		Females (n = 2)	26.76 ± 2.8	
***Cc16*$^{-/-}$ 24 wks CS 12 wks Vehicle**	Male (n = 3)	22.6 ± 1.6	0.12	Male (n = 3)	42.67 ± 7.7	0.49
	Females (n = 3)	20.77 ± 0.4		Females (n = 3)	49.43 ± 13.6	
**WT 24 wks CS 12 wks rhCC16**	Male (n = 4)	18.66 ± 1.5	0.42	Male (n = 4)	21.76 ± 3.7	0.84
	Females (n = 3)	17.82 ± 0.7		Females (n = 3)	23.37 ± 1.9	
***Cc16*$^{-/-}$ 24 wks CS 12 wks rhCC16**	Male (n = 3)	19.30 ± 0.9	0.25	Male (n = 4)	28.48 ± 10.3	0.94
	Females (n = 3)	18.34 ± 0.8		Females (n = 3)	29.0 ± 4.4	

§WT and *Cc16*$^{-/-}$ mice were exposed to air (5-6 mice/group) or CS (12-14 mice/group) for 24 weeks, and rhCC16 protein solution (75 µg of rhCC16; 6-7 mice/group) or vehicle (6 mice/group) was delivered thrice weekly by the intranasal route to CS-exposed mice for the last 12 weeks of the CS exposures. Sections of inflated and fixed lungs were stained with either Gill's stain and alveolar chord lengths as a measure of airspace size were quantified.

‡ WT and *Cc16*$^{-/-}$ mice were exposed to air or CS for 24 weeks, and rhCC16 protein solution (75 µg of rhCC16) or vehicle was delivered thrice weekly by the intranasal route to CS-exposed mice for the last 12 weeks of the CS exposures. Sections of inflated and fixed lungs were stained with Masson Trichrome stain (which stains extracellular matrix [ECM] proteins blue) and the thickness of the layer of ECM deposited around the small airways was quantified.

*Data were analyzed using one-way ANOVAs followed by pair-wise testing with Student's t tests.

Table 2. Respiratory mechanics Parameters in WT and *Cc16*$^{-/-}$ exposed to 6 months of Air or Cigarette Smoke (CS), treated with rhCC16 or vehicle.

Parameter	6 Month AIR		6 Month CS				$P^{¥}$
			Vehicle-treated		rhCC16-treated		
	WT (n=9)	*Cc16*$^{-/-}$ (n=9)	WT (n=14)	*Cc16*$^{-/-}$ (n=13)	WTC (n=15)	*Cc16*$^{-/-}$ (n=16)	
	Median (IQR)	Median (IQR)	Median (IQR)	Median (IQR)	Median (IQR)	Median (IQR)	
**Tissue Resistance**	3.563 (3.22 - 4.46)	3.501 (3.37 - 3.69)	3.880 (3.52 - 4.16)	3.527 (3.34 - 3.92)	3.504 (3.20 - 4.02)	3.515 (3.06 - 3.94)	0.458
**Tissue Elastance**	19.590 (19.15 - 21.18)	19.288 (17.56 - 20.06)	19.749 (17.04 - 21.39)	18.887 (18.10 - 20.00)	19.662 (17.84 - 20.16)	19.220 (18.07 - 20.16)	0.848
**Quasi-Static Compliance (*Cst* PVP)**	0.081 (0.07 – 0.08)	0.077* (0.07 – 0.08)	0.081 (0.07 – 0.08)	0.082 (0.07 – 0.08)	0.085 (0.07 - 0.08)	0.083[&] (0.08 – 0.09)	0.174

WT and *Cc16*$^{-/-}$ mice were exposed to air (9 mice/group) or CS (29 mice/group) for 6 months, treated with rhCC16 (15-16 mice/group) or vehicle (13-14 mice/group) for the last 12 weeks of CS-exposure. Respiratory mechanics were evaluated using a Flexivent device. Data are expressed in medians and interquartile ranges (IQR) for data that are not normally distributed.

[¥]Data were analyzed with One-way ANOVAs followed by pair-wise comparisons using Mann Whitney U tests.

* $P < 0.05$ when comparing WT vs. *Cc16*$^{-/-}$ mice exposed to air for 6 months.

[&]$P < 0.05$ when comparing CS exposed vehicle-treated *Cc16*$^{-/-}$ mice vs. rhCC16-treated *Cc16*$^{-/-}$ mice.

Table E3. Gender differences in absolute leukocyte and subset counts from bronchoalveolar lavage in cigarette smoke-exposed WT and *Cc16*$^{-/-}$ mice treated with rhCC16 protein solution.

	Sex	Leukocytes§	*P**	Macrophages§	*P**	PMN§	*P**	Lymphocytes§	*P**
**WT Air (n = 7)**	Male (n = 4)	10.26 ± 3.1	0.50	10.09 ± 3.1	0.53	0.03 ± 0.06	0.23	0.14 ± 0.03	0.09
	Females (n = 3)	8.9 ± 0.6		8.8 ± 0.6		0.01 ± 0.02		0.06 ± 0.06	
***Cc16*$^{-/-}$ Air (n = 8)**	Male (n = 4)	13.04 ± 3.1	0.75	12.7 ± 3	0.76	0.15 ± 0.14	0.98	0.18 ± 0.16	0.62
	Females (n = 4)	13.7 ± 2.6		13.3 ± 2.5		0.14 ± 0.01		0.22 ± 0.1	
**WT 24 wks CS 12 wks Vehicle (n = 10)**	Male (n = 5)	19.6 ± 79	0.52	17.7 ± 7.2	0.39	1.02 ± 1.2	0.54	0.88 ± 0.7	0.31
	Females (n = 5)	22.5 ± 5.8		21.1 ± 4.6		0.87 ± 0.9		0.53 ± 0.3	
***Cc16*$^{-/-}$ 24 wks CS 12 wks Vehicle (n = 13)**	Male (n = 5)	26.08 ± 4.6	0.07	22.5 ± 3.6	0.10	2.07 ± 1.4	0.43	1.54 ± 0.8	0.41
	Females (n = 8)	32.5 ± 6.2		27.6 ± 5.7		2.71 ± 1.4		2.19 ± 1.6	
**WT 24 wks CS 12 wks rhCC16 (n = 10)**	Male (n = 5)	17.5 ± 1.6	0.90	16.7 ± 1.4	0.67	0.5 ± 0.2	0.09	0.3 ± 0.2	0.11
	Females (n = 5)	17.7 ± 3.6		17.4 ± 3.5		0.17 ± 0.2		0.01 ± 0.1	
***Cc16*$^{-/-}$ 24 wks CS 12 wks rhCC16 (n = 15)**	Male (n = 6)	17.28 ± 3.7	0.85	16.0 ± 2.9	0.87	0.65 ± 0.3	0.12	0.61 ± 0.7	0.59
	Females (n = 9)	17.9 ± 6.8		16.5 ± 6.5		1.05 ± 0.5		0.3 ± 0.17	

WT and *Cc16*$^{-/-}$ mice were exposed to air (7-8 mice/group) or CS for 24 weeks (20-28 mice/group). CS-exposed mice were treated with rhCC16 protein solution (75 µg of rhCC16; 10-15 mice/group) or vehicle (13-15 mice/group) thrice weekly for the last 12 weeks of the exposures and bronchoalveolar lavage (BAL) was performed. BAL total leukocytes, macrophages, PMNs and lymphocytes were counted, and expressed as mean ± SD (standard deviation).

§All results are expressed as x 10^4 cells.

*Data were analyzed using one-way ANOVAs followed by pair-wise testing with Student's t tests.

Table E4. Antibodies used for lymphocyte subset quantification.

Fluorochrome	Antigen	Isotype	Clone	Company
FITC	CD4	Rat IgG2b, κ	RM4-4	BioLegend (San Diego, CA)
PE	Tim1	Rat IgG2b, κ	RMT1-4	BioLegend (San Diego, CA)
PerCP	CD8	Rat IgG2a, κ	53-6.7	BioLegend (San Diego, CA)
APC	PD-1	Rat IgG2a, κ	29F.1A12	BioLegend (San Diego, CA)
APC-Cy7	CD45	Rat IgG2b, κ	30-F11	BioLegend (San Diego, CA)
PE-Cy7	Tim3	Rat IgG1, κ	B8.2C12	BioLegend (San Diego, CA)
BV421	CD19	Rat IgG2a, κ	6D5	BioLegend (San Diego, CA)
AF700	CD45	Rat IgG2b, κ	30-F11	BioLegend (San Diego, CA)
FITC	CD25	Rat IgG2b, κ	3C7	BioLegend (San Diego, CA)
PE	ST2	Rat IgG2a, κ	DIH9	BioLegend (San Diego, CA)
PerCP	Thy1.2/CD90.2	Rat IgG2b, κ	30-H12	BioLegend (San Diego, CA)
APC-Cy7	CD45	Rat IgG2b, κ	30-F11	BioLegend (San Diego, CA)
PE-Cy7	TIGIT	Mouse IgG1, κ	1G9	BioLegend (San Diego, CA)
BV421	Foxp3	Rat IgG2a, κ	FJK-16s	eBioscience (San Diego, CA)
AF700	CD4	Rat IgG2a, κ	DIH9	BioLegend (San Diego, CA)

The table shows the isotype of the antibodies and fluorochromes that were used to quantify lymphocyte subsets in enzymatic digests of lungs from WT and *Cc16*$^{-/-}$ mice.

Table E5. List of primer sequences used to quantify the expression of chemokines, cytokines, mucins, and markers of senescence or Wnt-beta catenin activation in murine lungs

**Gene**	**Forward primer**	**Reverse primer**	**Product Length (nucleotides)**
Ccl-2	TTAAAAACCTGGATCGGAACCAA	GCATTAGCTTCAGATTTACGGGT	121
Ccl-3	TTCTCTGTACCATGACACTCTGC	CGTGGAATCTTCCGGCTGTAG	100
Ccl-5	GCTGCTTTGCCTACCTCTCC	TCGAGTGACAAACACGACTGC	104
Tnf-α	CCCTCACACTCAGATCATCTTCT	GCTACGACGTGGGCTACAG	61
Il-6	TAGTCCTTCCTACCCCAATTTCC	TTGGTCCTTAGCCACTCCTTC	76
Il-10	GCTCTTACTGACTGGCATGAG	CGCAGCTCTAGGAGCATGTG	105
Tgf-β	CTCCCGTGGCTTCTAGTGC	GCCTTAGTTTGGACAGGATCTG	133
Muc1	GGCATTCGGGCTCCTTTCTT	TGGAGTGGTAGTCGATGCTAAG	132
Muc5ac	GTGGTTTGACACTGACTTCCC	CTCCTCTCGGTGACAGAGTCT	103
Muc5b	GTGGCCTTGCTCATGGTGT	GGACGAAGGTGACATGCCT	139
Foxj1	ACCCTACTCCTATGCCACTCTCAT	TGCATGGCGGAAGTAGCAGAAGTT	24
Tcf7	AGCTTTCTCCACTCTACGAACA	AATCCAGAGAGATCGGGGGTC	115
Wisp2	CGCTGTGATGACGGTGGTTT	CCTGGCACCTGTATTCTCCTG	101
p16	CGCAGGTTCTTGGTCACTGT	TGTTCACGAAAGCCAGAGCG	127
p21	TGGAAACCATGATGCTTACGTT	GAAGCCCACTTTGCCATCTC	182
Sirtuin	GCTGACGACTTCGACGACG	TCGGTCAACAGGAGGTTGTCT	101
Ppia	GAGCTGTTTGCAGACAAAGTTC	CCCTGGCACATGAATCCTGG	125

The table shows the sequences of the primers that were used to perform quantitative real-time RT-PCR assays to quantify the expression of chemokines (*Ccl-2*, *Ccl-3*, and *Ccl-5*), mediators of inflammation (*Tnf-α*, *Il-6*, *Il-10*, and *Tgf-β*), mucins (*Muc1*, *Muc5ac*, and *Muc5b*), ciliary motility (*Foxj1*), Wnt-beta catenin activation (*Tcf7* and *Wisp2*) and senescence markers (*p16, p21, and sirtuin*). Peptidylprolyl isomerase A (*Ppia*) was used as the housekeeping gene.

Figure E1

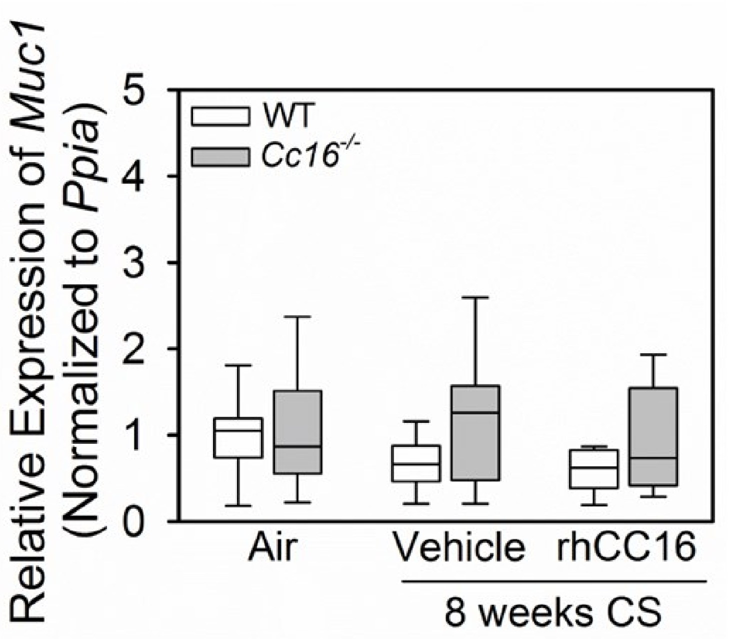

Supplemental Figure E1: ***Treating CS-exposed WT or Cc16$^{-/-}$ mice with rhCC16 protein solution does not affect Muc1 expression in their lungs.*** WT and *Cc16$^{-/-}$* mice were exposed to air (7 mice/group) or CS for 8 weeks (15-17 mice/group). CS-exposed mice were treated thrice weekly with rhCC16 protein solution (8 mice/group) or vehicle (7-9 mice/group) for the last 4 weeks of the 8 week CS exposure. Real-time PCR was used to quantify the expression of *Muc1* in whole lung samples. Boxes in the box-plots show the medians and 25th and 75th percentiles, and whiskers show the 10th and 90th percentiles. The Shapiro-Wilk test was used to determine whether the data were normally distributed, and Brown-Forsythe test was used to assess equal variance. Data were analyzed using one-way ANOVAs followed by pair-wise testing with Mann-Whitney U tests.

Figure E2

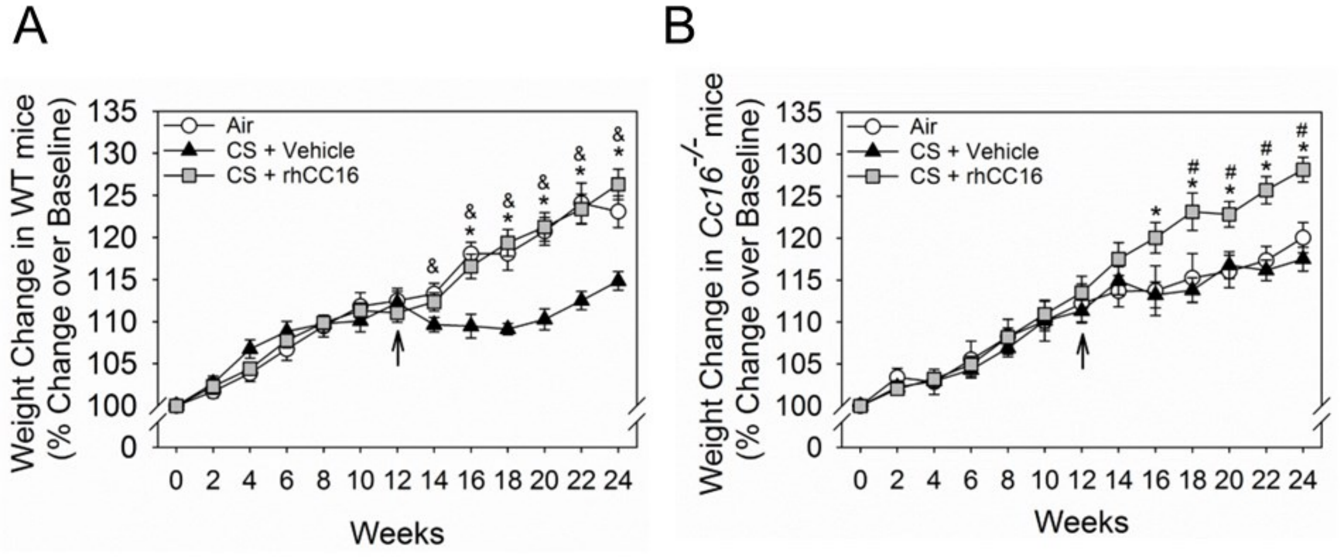

Supplemental Figure E2: ***Treating CS-exposed WT or Cc16$^{-/-}$ mice with rhCC16 protein solution increases their body weight.*** WT and *Cc16$^{-/-}$* mice were exposed to air (7-8 mice/group) or CS for 24 weeks (20-28 mice/group). CS-exposed mice were treated with rhCC16 protein solution (75 µg of rhCC16; 10-15 mice/group) or vehicle (10-13 mice/group) thrice weekly for the last 12 weeks of the 24 week CS exposure. Weight was recorded at baseline and every 2 weeks thereafter. ANOVA for repetitive measures was used to analyze changes in body weight from baseline values, followed by pair-wise testing 2-sided Student's t-tests. Data are mean ± SEM. In **A-B**, * indicates $P < 0.05$ for CS-exposed and rhCC16-treated mice versus CS-exposed and vehicle-treated mice at the same time point. In **A**, & indicates $P < 0.05$ for CS-exposed and vehicle-treated mice versus air-exposed mice at the same time point. In **B**, # indicates $P < 0.05$ for CS-exposed and rhCC16-treated mice versus air-exposed mice at the same time point. The arrow indicates the time point when the treatments with vehicle or rhCC16 protein solution were initiated.

Figure E3

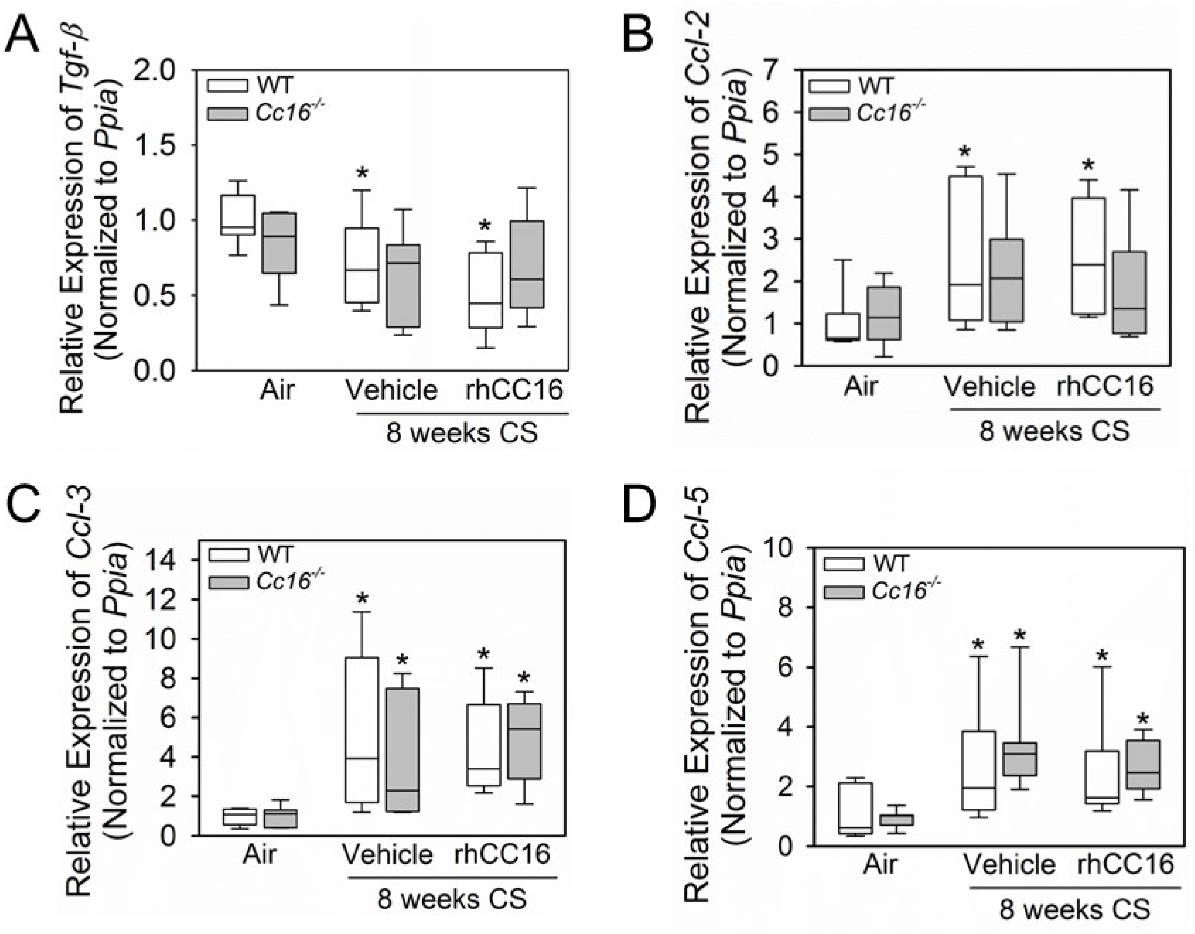

Supplemental Figure E3: ***Treating CS-exposed WT or Cc16$^{-/-}$ mice with rhCC16 protein solution has no effect on the expression of Tgf-β or several chemokines in their lungs.*** WT and *Cc16$^{-/-}$* mice were exposed to air (7 mice/group) or CS for 8 weeks (15-17 mice/group). CS-exposed mice were treated thrice weekly with rhCC16 protein solution (8 mice/group) or vehicle (7-9 mice/group) for the last 4 weeks of the 8 week CS exposure. Real-time PCR was used to quantify the expression of *Tgf-β* (**A**), *Ccl-2* (**B**), *Ccl-3* (**C**), and *Ccl-5* (**D**) in whole lung samples. Boxes in the box-plots show the medians and 25th and 75th percentiles, and whiskers show the 10th and 90th percentiles. The Shapiro-Wilk test was used to determine whether the data were normally distributed, and Brown-Forsythe test was used to assess equal variance. Data were analyzed using one-way ANOVAs followed by pair-wise testing with Mann-Whitney U tests. Asterisks indicate $P < 0.05$ versus air-exposed mice belonging to the same genotype.

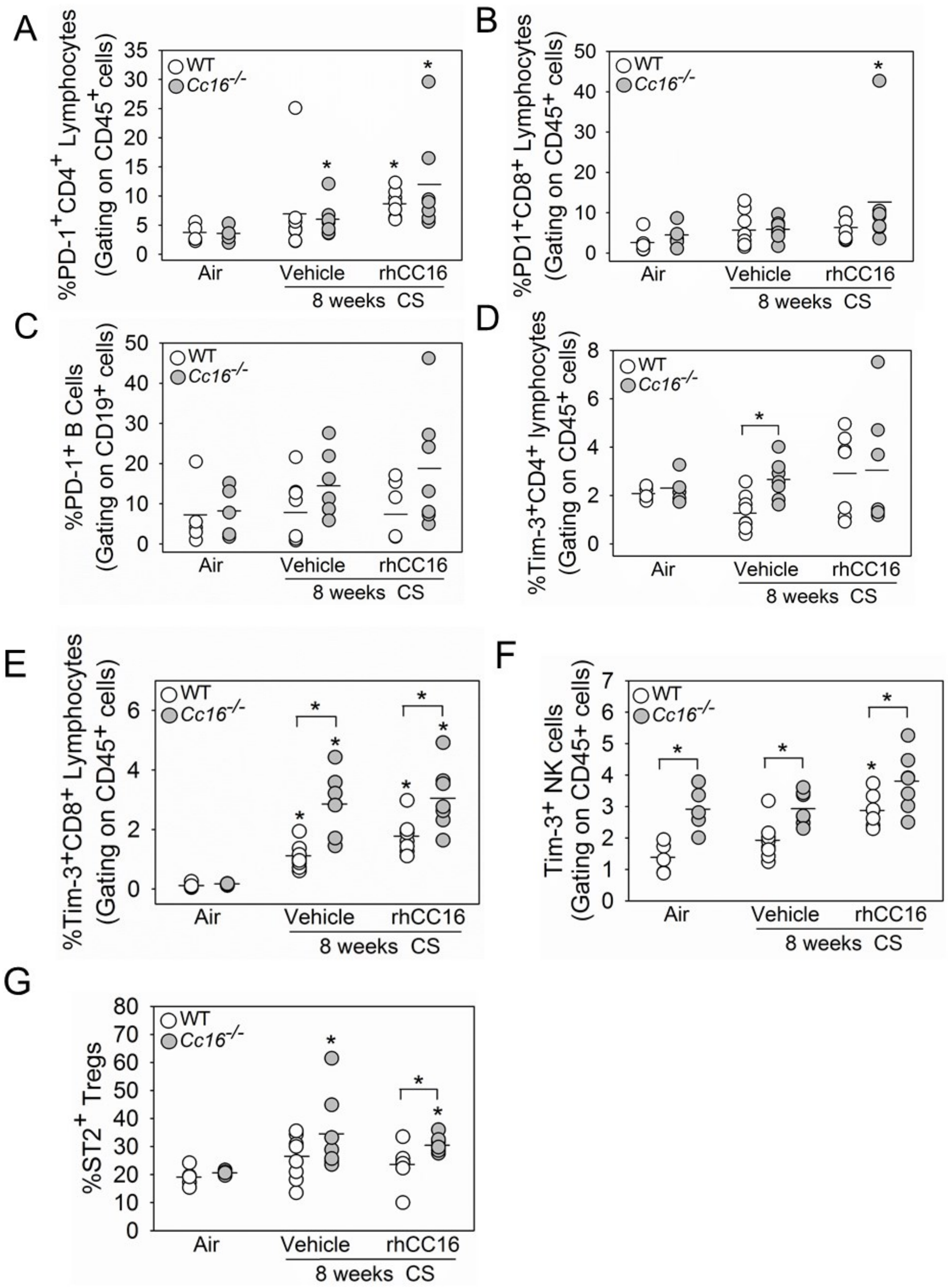

Supplemental Figure E4: *Treating CS-exposed WT or Cc16$^{-/-}$ mice with rhCC16* protein solution does not alter some *lymphocyte subset frequencies in enzymatic lung digest samples.* WT and *Cc16$^{-/-}$* mice were exposed to air (5 mice/group) or CS for 8 weeks (14-15 mice/group), and CS-exposed mice were treated with rhCC16 protein solution (75 µg of rhCC16; 7 mice/group) or vehicle (7-8 mice-group) thrice weekly for the last 4 weeks of the 8 week CS exposure. Lungs were enzymatically digested, and lymphocyte subsets were quantified by immunostaining lung cells for markers of lymphocyte subsets

and lymphocyte activation, and cells were analyzed by flow cytometry. **A**: PD-1^+CD4^+ lymphocytes (gating on CD45^+ cells); **B**: PD-1^+CD8^+ lymphocytes (gating on CD45^+ cells); **C**: PD1^+ B cells (gated on CD19^+ lymphocytes); **D**:Tim-3^+CD4^+ lymphocytes (gating on CD45^+ cells); **E**: Tim-3^+CD8^+ lymphocytes (gating on CD45^+ cells); **F**: Tim-3^+NK cells (gated on CD19^+ lymphocytes); and **G**: ST2^+Tregs (gating on CD4^+CD25^+Foxp3^+ lymphocytes). The horizontal bars in the dot-plots represent the mean values. Data were analyzed using one-way ANOVAs followed by pair-wise testing with Mann-Whitney U tests. Asterisks indicate $P < 0.05$ versus air-exposed mice belonging to the same genotype or the group indicated.

Figure E5

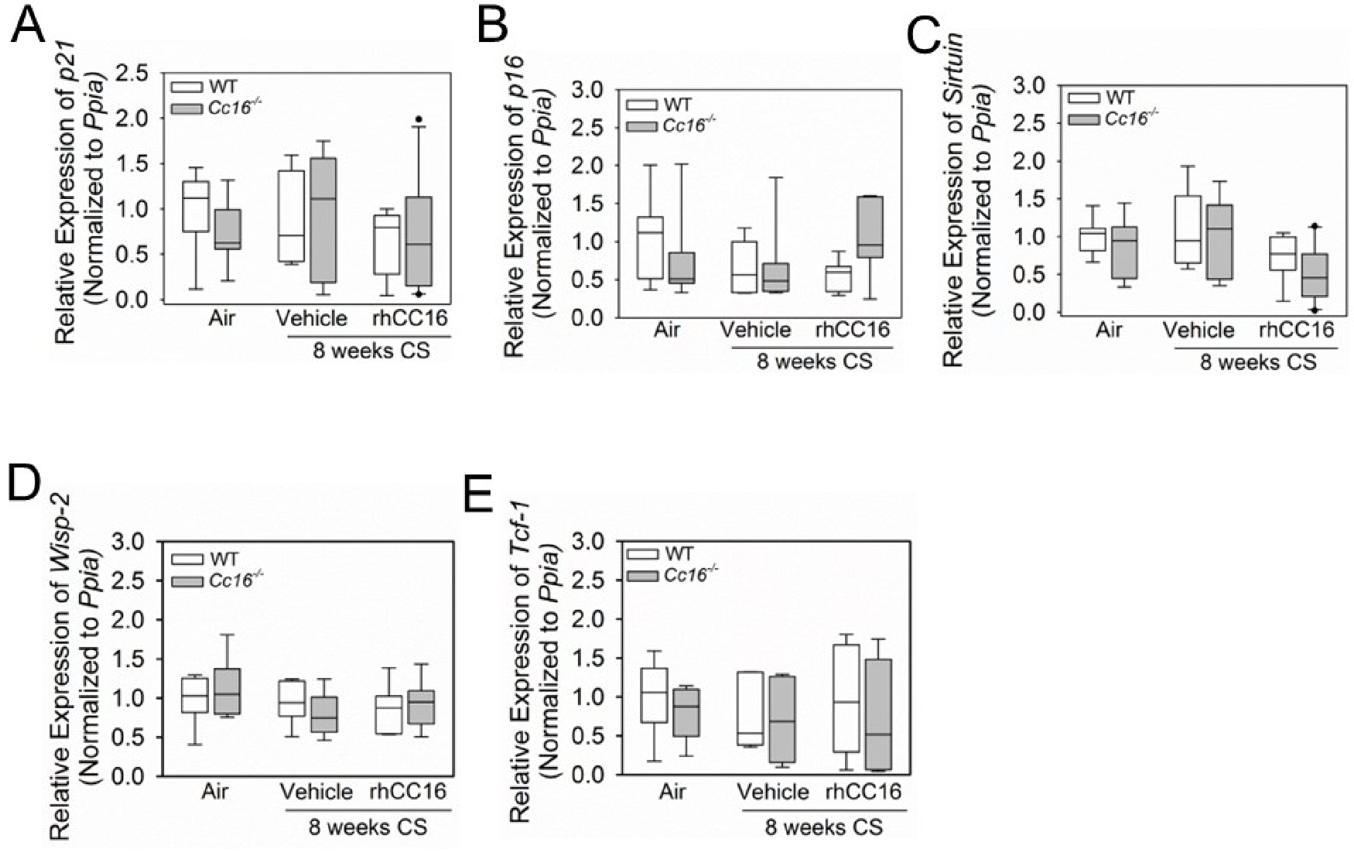

Supplemental Figure E5: ***Treating CS-exposed WT or Cc16$^{-/-}$ mice with rhCC16 protein solution does not alter the expression of markers of senescence or activation of the Wnt-beta catenin pathway in lung samples.*** WT and *Cc16$^{-/-}$* mice were exposed to air (7 mice/group) or CS for 8 weeks (15-17 mice/group). CS-exposed mice were treated thrice weekly with rhCC16 protein solution (8 mice/group) or vehicle (7-9 mice/group) for the last 4 weeks of the 8 week CS exposure. Real-time PCR was used to quantify the expression of *p21* (**A**), *p16* (**B**), *Sirtuin* (**C**), *Wisp-2* (**D**) and *Tcf-1* (**E**). Boxes in the box-plots show the medians and 25th and 75th percentiles, and whiskers show the 10th and 90th percentiles. The Shapiro-Wilk test was used to determine whether the data were normally distributed, and Brown-Forsythe test was used to assess equal variance. Data were analyzed using one-way ANOVAs followed by pair-wise testing with Mann-Whitney U tests.

Figure E6

A

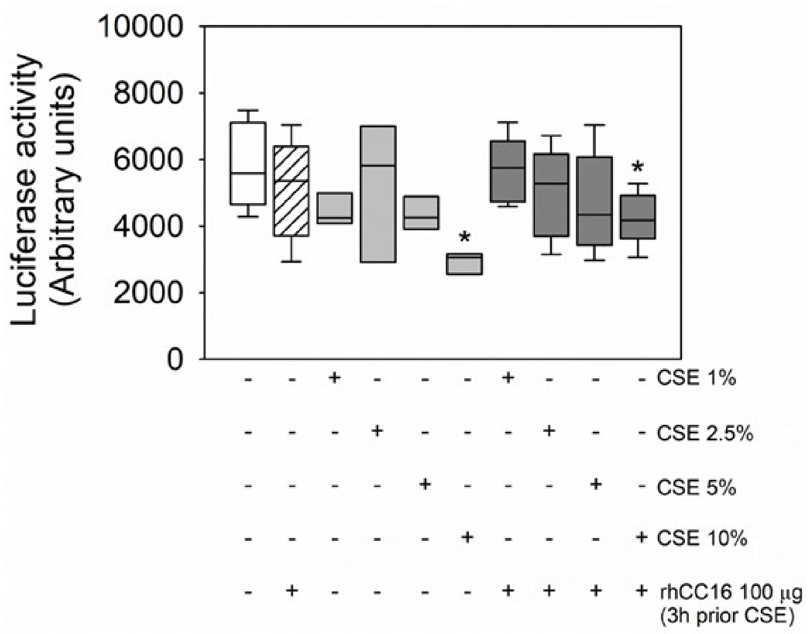

Figure E6. Cigarette smoke extract [CSE] doesn't activate NF-κB in luciferase reporter A549 cells. NF-κB luciferase reporter A549 cells were cultured to >80% confluence in complete media. In **A**, NF-κB luciferase reporter A549 cells were incubated with rhCC16 protein solution for 3 h, and then 1-10% CSE was added and cells were incubated for a further 24 h. Cells were then lyzed and luciferase activity was measured. Boxes in the box-plots show the medians and 25th and 75th percentiles, and whiskers show the 10th and 90th percentiles. Data were analyzed using one-way ANOVAs followed by pair-wise testing with Mann-Whitney U tests. Asterisks indicate *P* < 0.05 versus the negative control (no CSE and no rhCC16).

Figure E7

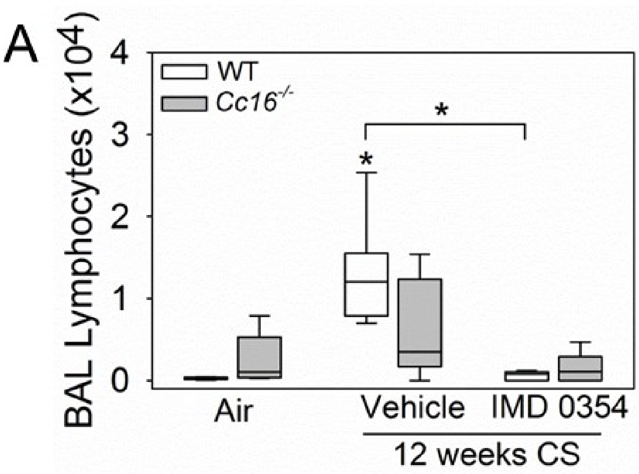

Supplemental Figure E7: *Treating CS-exposed Cc16$^{-/-}$ mice with IMD0354 solution reduces their exaggerated pulmonary inflammatory response to CS.* In **A** and **B**, WT and *Cc16$^{-/-}$* mice were exposed to air (3-5 mice/group) or CS for 12 weeks (10-12 mice/group), and CS-exposed mice were treated once daily with a solution of IMD0354 (6 mg/kg body weight, 5-6 mice/group) or vehicle via the intraperitoneal route during the last 6 weeks of the 12 week CS exposures (5-6 mice/group). Bronchoalveolar lavage (BAL) was performed and BAL lymphocytes were counted. Boxes in the box-plots show the medians and 25th and 75th percentiles, and whiskers show the 10th and 90th percentiles. Data were analyzed using one-way ANOVAs followed by pair-wise testing with Mann-Whitney U tests. Asterisks indicate $P < 0.05$ versus air-exposed mice belonging to the same genotype or the group indicated.
